# Programmable Paracrine‐Mimetic Microneedle System for Temporal Regulation of Cardiac Repair

**DOI:** 10.1002/advs.77101

**Published:** 2026-08-18

**Authors:** Yichen Dai, Pengchong Du, Zhaoyang Wang, Surilige, Rangga Fokta, Renjian Tan, Xiqing Zhao, Shuoyi Zhang, Yuecheng Wang, Yixin Zhang, Ziliang Fu, Rui Gong, Cheng Jiang, Yisheng He, Qiguang Wang, Renke Li, Khoon Lim, Junnan Tang, Xiaolin Cui

**Affiliations:** ^1^ School of Medicine The Chinese University of Hong Kong Shenzhen Guangdong China; ^2^ CUHKSZ‐Dalian Practical Biotechnology Joint Laboratory for Biomedicine The Chinese University of Hong Kong Shenzhen Guangdong China; ^3^ Department of Cardiology The First Affiliated Hospital of Zhengzhou University Henan China; ^4^ Henan Province Key Laboratory of Cardiac Injury and Repair Zhengzhou Henan China; ^5^ Henan Province Clinical Research Center For Cardiovascular Diseases Zhengzhou Henan China; ^6^ State Key Laboratory of Metabolic Dysregulation and Prevention and Treatment of Esophageal Cancer, Tianjian Laboratory of Advanced Biomedical Sciences Academy of Medical Sciences Zhengzhou Henan China; ^7^ School of Medical Sciences University of Sydney Sydney New South Wales Australia; ^8^ College of Biomedical Engineering Sichuan University Chengdu Sichuan China

**Keywords:** cardiac repair, growth factors, myocardial infarction, paracrine signaling

## Abstract

Dynamic paracrine signaling governs tissue repair, yet replicating this temporal complexity using acellular systems remains challenging. While recent advances include microcapped microneedle patches and the TIMED platform, these systems face limitations in customizability, release duration, and fabrication simplicity. Here, this study reports a programmable microneedle platform that mimics the temporal paracrine signaling logic of stem cell therapy by generating phase‐biased, kinetically staggered, and partially overlapping availability of TGF‐β, IGF‐1, and VEGF through a tris(2,2'‐bipyridyl)dichlororuthenium(II) hexahydrate/sodium persulfate (Ru/SPS)‐modulated tyramine crosslinked hydrogel network. This system demonstrates enhanced advantages, including straightforward tunability of release kinetics via Ru/SPS concentration adjustment, extended release duration up to ∼60 days, and a simplified fabrication process. In myocardial infarction models, this stem cell‐inspired temporal release promoted anti‐inflammatory, anti‐apoptotic, and pro‐angiogenic responses, restoring ventricular function. Direct schedule‐control experiments further showed that the tested TGF‐β/IGF‐1/VEGF program outperformed 7‐ and 28‐ day simultaneous‐window delivery and two order‐swapped 7/14/28‐day programs across functional, structural, angiogenic, and cardiomyocyte‐proliferation readouts. This strategy establishes a generalizable framework for engineering time‐coded therapeutic materials that emulate biological signaling programs.

## Graphical abstract

Temporal signaling is encoded into a programmable paracrine‐mimetic microneedle patch via tris(2,2'‐bipyridyl)dichlororuthenium(II) hexahydrate/sodium persulfate‐tuned hydrogel microparticles that deliver TGF‐β, IGF‐1, and VEGF over phase‐matched 7‐, 14‐, and 28‐day windows. In myocardial infarction models, this sequence suppresses inflammation, apoptosis, and fibrosis, enhances revascularization, and restores cardiac function, outperforming simultaneous and order‐swapped schedules and highlighting the therapeutic power of timing.

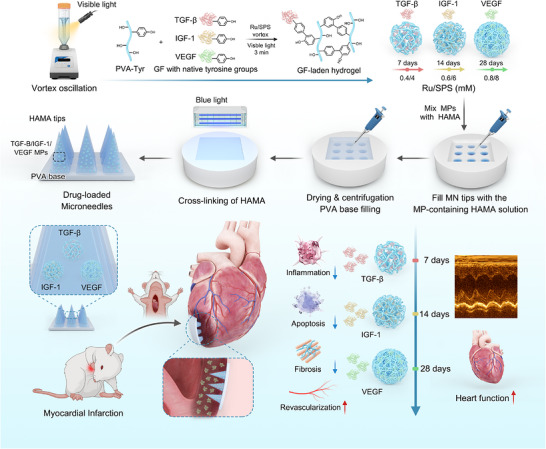

## 1 Introduction

Restoring the intricate signaling dynamics that govern tissue repair remains one of the grand challenges of regenerative medicine. For instance, following myocardial infarction (MI), the heart undergoes a tightly orchestrated sequence of inflammatory resolution, reparative remodeling, and neovascularization [1, 2, 3, 4, 5, 6]. Each stage is regulated by a transient yet precisely timed cascade of paracrine factors, including transforming growth factor‐β (TGF‐β), insulin‐like growth factor‐1 (IGF‐1), and vascular endothelial growth factor (VEGF). This temporal choreography determines whether the heart heals adaptively or progresses toward fibrosis and failure. However, conventional therapies—whether pharmacological or interventional—fail to emulate this spatiotemporal complexity, resulting in suboptimal or inconsistent clinical outcomes [7, 8].

Stem cell therapy has long been considered a promising strategy for cardiac repair because transplanted cells can secrete bioactive factors (e.g., TGF‐β, IGF‐1, and VEGF) that modulate inflammation, apoptosis, and angiogenesis in a stage–dependent manner [6, 9, 10, 11, 12, 13, 14, 15, 16, 17, 18, 19]. Yet, the poor survival of transplanted cells and the unpredictable nature of their paracrine activity limit reproducibility and safety [9]. Moreover, storage/shipping difficulty, regulatory barriers, and tumorigenic risks associated with stem cell‐based interventions have hindered their translation to clinical practice [9, 20, 21, 22, 23, 24, 25]. Consequently, there is a growing shift toward acellular approaches that can replicate the functional essence of stem cell paracrine signaling, not through living cells, but through intelligent materials capable of delivering multiple biomolecules in a programmable and biologically meaningful sequence.

Recombinant growth factors (GFs) such as TGF‐β, IGF‐1, and VEGF have each shown therapeutic potential in experimental MI models by modulating inflammation, preventing cardiomyocyte apoptosis, and promoting angiogenesis [26, 27, 28]. However, clinical translation has been hampered by two key limitations. First, these proteins have extremely short half‐lives in vivo, ranging from minutes to hours, resulting in rapid clearance and poor tissue retention [29, 30, 31]. Second, most existing delivery systems rely on static or single‐factor release, which cannot recapitulate the dynamic, time‐encoded interplay of multiple factors required for functional regeneration [32, 33]. The ability to control not only what biomolecules are released but also when and for how long they act is therefore critical to reproducing the natural healing timeline of the myocardium.

Recent advances in hydrogel chemistry and microneedle (MN) technologies offer new opportunities for localized and minimally invasive cardiac drug delivery [34]. MN patches can penetrate the epicardial surface and deposit therapeutic cargos directly into the infarcted myocardium, reducing systemic loss and enhancing local bioavailability [35, 36]. Meanwhile, the development of stimuli‐responsive hydrogels enables tunable degradation and release kinetics [37, 38]. However, existing MN systems predominantly achieve sustained or burst release profiles rather than orchestrated, multiphase temporal delivery [28, 39, 40, 41]. Notably, while most recently developed systems like the microcapped MN patch [42] and the TIMED platform [43] have made attempts in sequential drug delivery, they face limitations in customizability, release duration, and fabrication complexity. In addition, their drug set and temporal logic are not specifically framed as a paracrine mimic of stem cell‐driven cardiac repair. Moreover, while both studies demonstrate therapeutic efficacy, mechanistic analyses are limited mainly to histology and a focused panel of markers, leaving the global remodeling of extracellular matrix (ECM), adhesion, and metabolic pathways under time‐programmed therapy less explored [42, 43].

**SCHEME 1:**
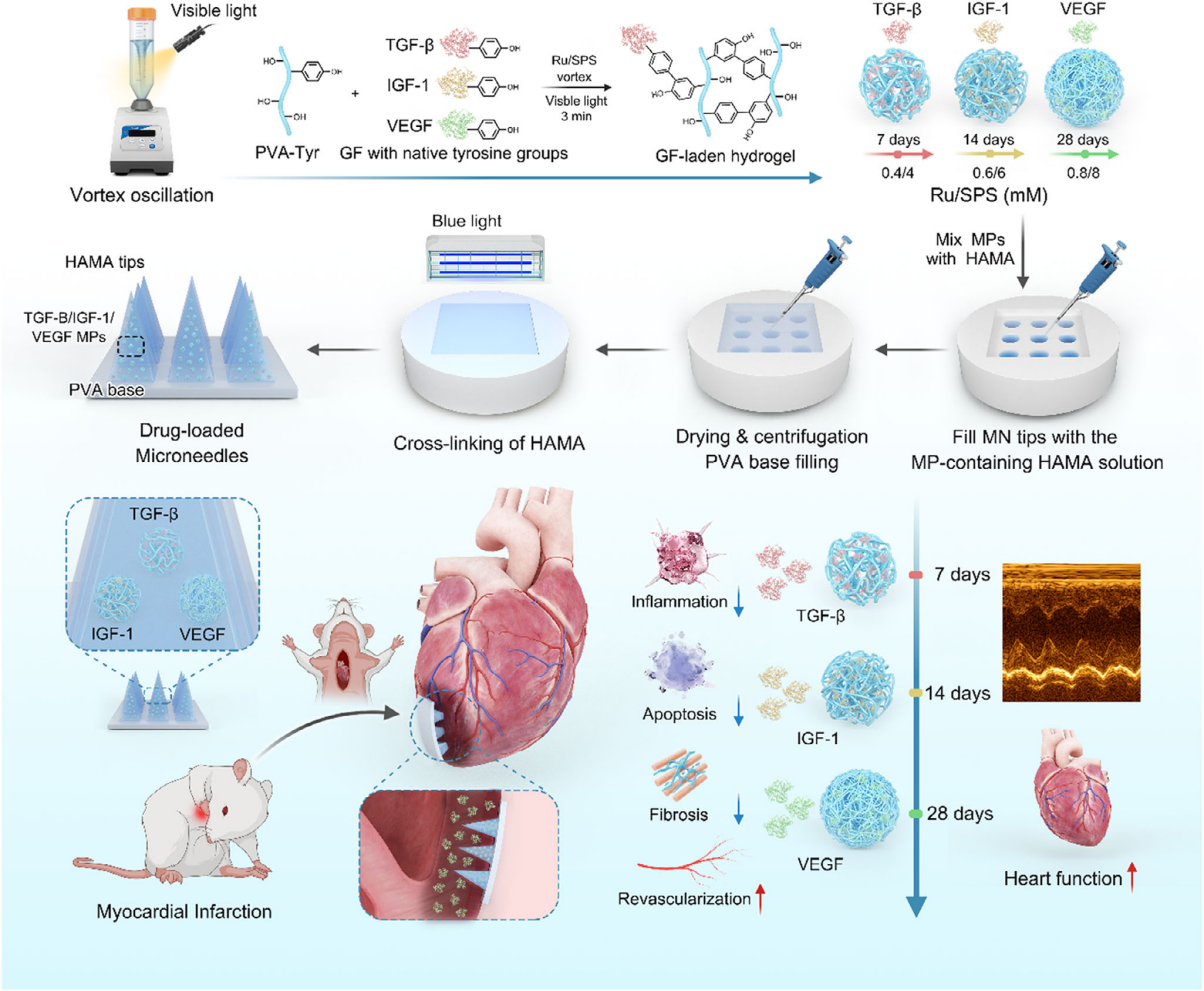
Schematic diagram of the programmable paracrine‐mimetic microneedle system for the temporal treatment of myocardial infarction.

Here, we present a programmable paracrine‐mimetic MN (PM‐MN) system that recapitulates the temporal logic of stem cell‐mediated cardiac repair (Scheme 1). Our design aimed to mimic the staged paracrine secretion pattern of stem cell therapy, offering key advantages over existing systems, including easy and enhanced temporal tunability, extended release duration up to ∼60 days, and a simplified fabrication process that avoids multimaterial complexity. Our design integrates two complementary material components: a biocompatible hyaluronic acid methacrylate (HAMA) matrix forming the MN tips for direct myocardial penetration, and tyramine‐modified poly(vinyl alcohol) (PVA‐Tyr) microparticles (MPs) serving as programmable reservoirs for GFs [44, 45]. By modulating the tris(2,2'‐bipyridyl)dichlororuthenium(II) hexahydrate/sodium persulfate (Ru/SPS) photo‐initiated crosslinking density within the PVA‐Tyr network, we generated phase‐biased, kinetically staggered, and partially overlapping GF availability windows, with ordered dominant‐availability and release‐completion windows for TGF‐β, IGF‐1, and VEGF. These windows were selected to align with biologically relevant and overlapping phases of post‐MI repair and were directly benchmarked against simultaneous‐window and order‐swapped schedules in vivo.

The novelty of this study is different from conventional sustained‐release platforms and existing GF delivery system. Instead of maximizing retention time or cumulative dosage, we sought to program temporal information into the material itself, encoding a predefined biological sequence within its degradation kinetics. This represents a shift from passive drug delivery toward active temporal programming, a design principle inspired by developmental biology, where the timing of morphogen release dictates tissue fate [46]. By embedding this stem cell‐mimetic logic into synthetic materials, our PM‐MN platform bridges the gap between cell therapy and acellular bioengineering, offering a more controllable, reproducible, and clinically adaptable alternative to systems like the microcapped MN patch and the TIMED platform [42, 43]. In addition, beyond cardiac repair, this strategy offers a versatile blueprint for developing time‐encoded regenerative materials applicable to wound healing, neuroregeneration, and tissue homeostasis, where dynamic coordination of multiple biological cues is essential for functional recovery.

## 2 Results

### 2.1 Fabrication and Characterization of the MN Patch and PVA‐Tyr MPs

**FIGURE 1:**
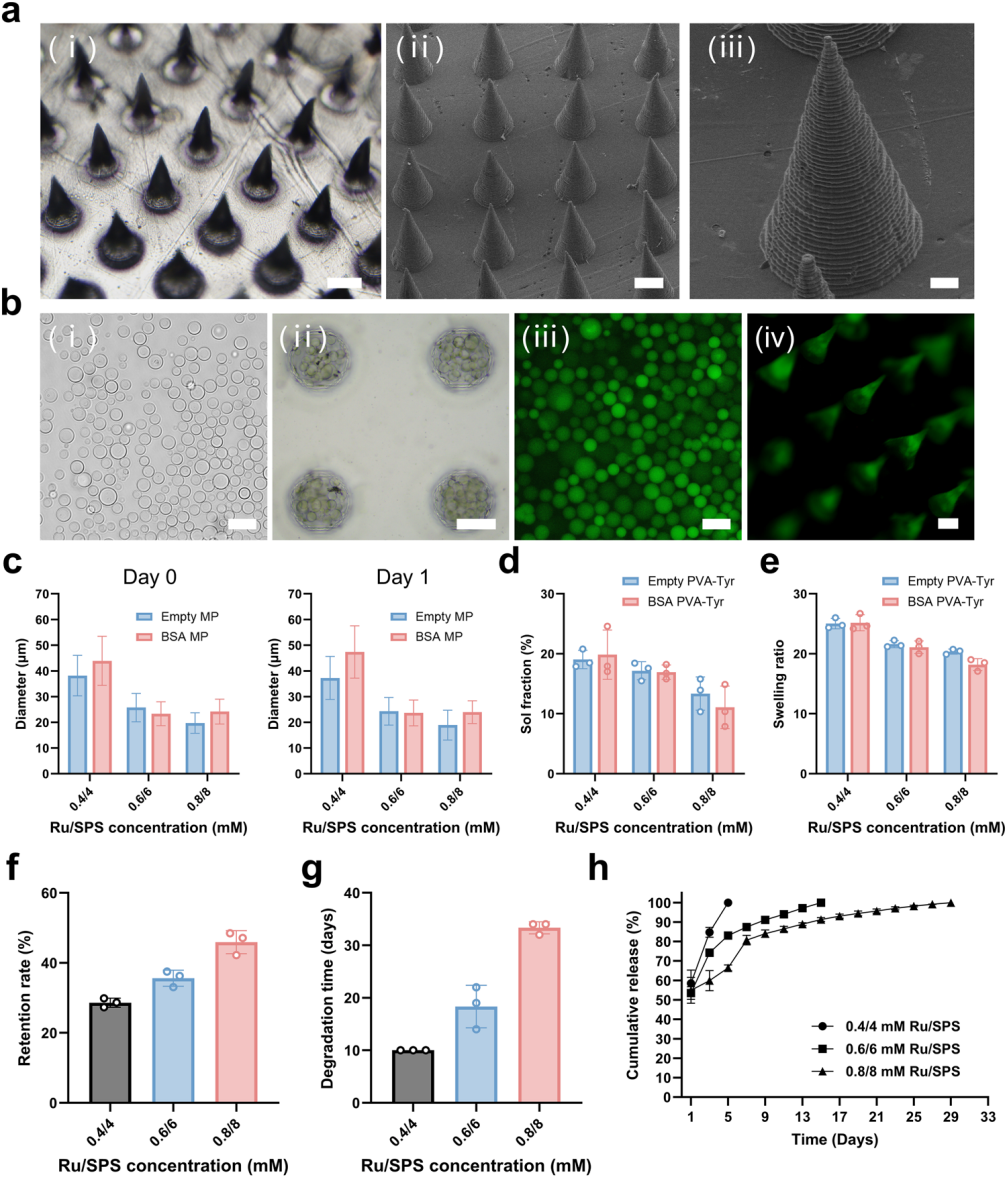
Characterization of HAMA‐MN patch and PVA‐Tyr MPs. (a) Representative images of the MN patch: (i) Representative optical and (ii) SEM images of the MN patch, (iii) enlarged SEM image of the MN patch. Scale bar = 200, 200, and 50 µm, respectively. (b) Representative images of (i) PVA‐Tyr MPs (0.8/8 mm Ru/SPS), (ii) MPs loaded into cavities of the MN patch, fluorescent image of (iii) BSA‐FITC loaded MPs and (iv) MN tips filled with BSA‐FITC loaded MPs. Scale bar = 50, 200, 50, and 200 µm, respectively. (c) Size of PVA‐Tyr MPs loaded with or without BSA (1 mg/mL) at day 0 and day 1; (d) sol fraction and (e) swelling ratio of PVA‐Tyr MPs with or without BSA (1 mg/mL); (f) retention rate, and (g) degradation time of PVA‐Tyr MPs with different Ru/SPS concentrations (0.4/4, 0.6/6, 0.8/8 mm) (*n* = 3). (h) Cumulative release profiles of BSA from the MN loaded with MPs (*n* = 3).

Hyaluronic acid methacrylate (HAMA, MW ∼150 kDa) has been well documented for its excellent biocompatibility and photo‐crosslinking ability. Previous studies have demonstrated the photocrosslinked HAMA (MW ∼150 kDa) hydrogel has sufficient mechanical strength to penetrate into the myocardium [36, 47, 48]. Thus, HAMA was selected for the fabrication of the MN tips to deliver pharmaceuticals into the inner site of the infarction area. PVA was further employed as the base/backing material of the MN because PVA and PVA‐based MN backings have been reported to exhibit good flexibility and biocompatibility, enabling conformal contact with curved tissue/skin surfaces [49, 50, 51]. The morphology of the MN patch was characterized using both inverted microscope (Figure 1a (i)) and scanning electron microscope (SEM) (Figure 1a (ii) and (iii)). As illustrated in Figure 1a, the MN tips exhibited a uniform conical geometry with a base diameter of 300 µm and a height of 550 µm. The spacing between adjacent tips was 600 µm, forming a 10 × 10 square array. To characterize the PVA‐Tyr macromer, the ^1^H‐nuclear magnetic resonance (NMR) spectroscopy was used to validate the presence of the tyramine group on the PVA backbone, which demonstrated the successful conjugation of tyramine group with the presence of characteristic proton peaks for the aromatic ring at δ = 6.5–7.2 ppm (Figure S1a). The tyramined degree was calculated as seven tyramine groups per PVA chain. Optical microscope image shows that PVA‐Tyr MPs (0.8/8 mm Ru/SPS) were sphere‐shaped and diameters ranged from 15 to 30 µm with the peak at around 20 µm (Figure 1b (i) and c), which were well‐suited for loading into the MN tips. MPs were successfully encapsulated into the cavities of the MN patch via centrifugation, evidenced by the optical image (Figure 1b (ii)) and bovine serum albumin (BSA)‐FITC‐labeled MPs (Figure 1b (iii) and (iv)). The mechanical strength of the MN patch was over 8 N (Figure S1b). Considering that the MN array contains 10 × 10 needles, this corresponds to an estimated average load of approximately 0.08 N per needle under full‐array contact. In our system, the MN is integrated with a soft, compliant, and relatively thick PVA base that enables intimate contact with the dynamic heart surface (Video S1, Supporting Information). During the compression test of the MN patch, deformation of this base layer buffers and redistributes the load, which masks abrupt local failure signals from individual needles. Therefore, the compression curve did not show a distinguishable fracture point that could be reliably assigned to single‐needle failure. Nevertheless, reported stiffness of rat myocardium is in the range of 18–55 kPa, indicating that the mechanical strength of the MN patch is compatible with myocardial insertion [36]. Furthermore, Masson staining of the infarct region after MN application revealed multiple distinct puncture cavities generated by the MNs (Figure S1c), directly confirming successful penetration of the cardiac tissue. The storage modulus (*G*′) and loss modulus (*G*″) of the bulk PVA‐Tyr hydrogel with different Ru/SPS concentrations (0.4/4, 0.6/6, and 0.8/8 mm) was also investigated (Figure S1d). It was demonstrated that both modulus reached a stable value after exposure to the light for around 10 s, indicating the gelation process was completed. Besides, tan δ (*G*″/*G*′) values of PVA‐Tyr hydrogel fabricated with different Ru/SPS concentrations (0.4/4, 0.6/6, and 0.8/8 mm) were much lower than 1 and exhibited a rising trend with the increasing Ru/SPS concentration, implying that the hydrogel stability improves as the cross‐linking density enhances. In addition, the SEM images indicated the porous structure of PVA‐Tyr MPs (Figure S2a).

To achieve temporal controlled release of GF, different Ru/SPS concentrations (0.4/4, 0.6/6, and 0.8/8 mm) were selected for MP fabrication to tune the cross‐linking density. The size of MPs loaded with or without BSA demonstrated no significant difference at both day 0 and day 1 (after swelling), with the 0.4/4 mm Ru/SPS condition demonstrating the largest diameter (Figure 1c). The sol fraction and swelling ratio of PVA‐Tyr MPs (Figure 1d,e) indicated that higher Ru/SPS concentrations increased cross‐linking efficacy, while protein loading did not affect these parameters. To characterize retention, degradation timeline, and release kinetics, we employed FITC‐labeled BSA as the model protein, given that previous studies have shown that hydrolysis‐driven degradation of the PVA‐Tyr network governs GF release kinetics and that the release behavior of different GFs from PVA‐Tyr hydrogel closely resembles that of BSA [44, 45]. As shown in Figure 1f,g, both retention rate and degradation time for MPs alone exhibited dose‐dependent increase with Ru/SPS concentrations (0.4/4 mm: 28.63% and 10 days; 0.6/6 mm: 35.62% and 18.33 days; 0.8/8 mm: 45.94% and 33.33 days). Because directly quantifying the time‐resolved mass loss of MP within the MN tips is challenging, the cumulative release kinetics of proteins (BSA) from the embedded MPs were used to demonstrate the release profile and provide a practical quantitative readout of the time‐dependent degradation progression beyond the initial phase (Figure 1h). The post‐initial degradation‐rate profile can be interpreted from the slope of the cumulative release curve (Figure 1h). Notably, although the 0.4/4 mm (concentrations of Ru/SPS) group exhibited the largest MP diameter, it showed the fastest degradation/release, whereas higher Ru/SPS groups, despite having smaller MPs, exhibited slower degradation and more prolonged release. This trend is opposite to what would be expected if MP size were the dominant factor controlling release, confirming that hydrolysis‐driven degradation plays the major role in GF release [44, 45]. Furthermore, the degradation timeline of MPs embedded within the MN patch (time to completely release GFs) (Figure 1h) (0.4/4 mm: 5 days; 0.6/6 mm: 15 days; 0.8/8 mm: 29 days) showed a little difference from MPs alone. Degradation time of PVA‐Tyr MPs with higher concentrations of Ru/SPS (1/10 and 2/20 mm) was also recorded, which was 40 and 63.33 days, respectively (Figure S2b). Thus, this programmable PM‐MN system possesses extensive potential to provide personalized treatment strategies adaptable to disease progression, with tailorable timespan and release profile (up to around 60 days) of multiple incorporated proteins.

The burst release of GFs (∼50%–60% within 24 h; Figure 1h) was attributed to the rapid diffusion of physically encapsulated GFs from the PVA‐Tyr MPs. Importantly, the three GF‐loaded MP populations were combined on an equal formulation‐input basis at a 1:1:1 ratio, defined by equal PVA‐Tyr macromer input during MP synthesis. This yielded a theoretical loading of 200 ng TGF‐β, 200 ng IGF‐1, and 200 ng VEGF per MN patch. Because formulation retention varied with the crosslinking condition, the estimated post‐rinse retained amounts were approximately 57.3, 71.2, and 91.9 ng per patch, respectively. The selected formulation is therefore interpreted as a processability‐constrained, literature‐guided, and biologically staged design, rather than an exhaustively optimized stoichiometric cocktail. The retained fractions were subsequently released with formulation‐dependent post‐burst kinetics, generating ordered dominant‐availability and release‐completion windows.

To further clarify the release mechanism, we analyzed the cumulative release profiles using a burst‐separated Weibull fitting approach, and the corresponding fitted curves are shown in Figure S3. The burst fraction at day 1 was comparable among the three formulations (0.4/4 mm: 58.5%; 0.6/6 mm: 53.5%; 0.8/8 mm: 54.9%), indicating that the early release phase was not strongly altered by Ru/SPS concentration. In contrast, the later sustained phase was markedly prolonged as Ru/SPS increased. Based on the experimental release curves, the time to 99% cumulative release increased from 4.87 days for 0.4/4 mm, to 14.30 days for 0.6/6 mm, and to 26.57 days for 0.8/8 mm, respectively.

Because the three formulations exhibited comparable day‐1 burst release but distinct post‐burst release‐completion times, the PM‐MN system is described here as generating phase‐biased, kinetically staggered, and partially overlapping GF availability windows, rather than three fully isolated release pulses. In this context, sequential release denotes the ordered dominant‐availability and release‐completion windows of TGF‐β, IGF‐1, and VEGF.

This analysis supports a biphasic release mechanism consisting of an initial burst release of physically accessible or weakly retained protein followed by a slower post‐burst phase dominated by matrix‐controlled release. Such behavior is consistent with recent literature showing that Weibull‐based analysis is well suited to polymeric release systems, especially when simple diffusion‐only models are insufficient and when the beginning of the profile is affected by burst release or lag‐time behavior [52, 53].

Together with the sol fraction, swelling, rheological, and degradation data, these results further support that increasing Ru/SPS increases the effective network density/stability of the PVA‐Tyr MPs and thereby mainly prolongs the post‐burst hydrolysis/relaxation‐controlled release phase, rather than substantially changing the initial burst fraction.

### 2.2 Anti‐inflammatory Effects of TGF‐β‐loaded MP on RAW 264.7

Dysregulated inflammatory cascades during the early phase post MI exacerbate myocardial injury and promote maladaptive left ventricular (LV) remodeling, ultimately predisposing to heart failure progression [54]. To mitigate early inflammation, TGF‐β was selected to be encapsulated into the MP and then loaded into the MN. The concentration of Ru/SPS was 0.4/4 mm to ensure the complete release in the initial 7 days, aligning with the reparative phase post‐MI. The anti‐inflammatory effects of released TGF‐β were evaluated in vitro using RAW 264.7 macrophages. Under pro‐inflammatory stimulation, RAW 264.7 cells polarize toward the M1 phenotype, exhibiting elevated expression of surface markers, inflammatory cytokines, and nitric oxide (NO) [55, 56]. Lipopolysaccharide (LPS), a major component of Gram‐negative bacterial outer membranes (e.g., *Porphyromonas gingivalis*), is commonly used to induce macrophage activation and inflammation [57]. Accordingly, RAW 264.7 cells were stimulated with 10 ng/mL LPS for 8 h to establish the in vitro inflammation model.

**FIGURE 2:**
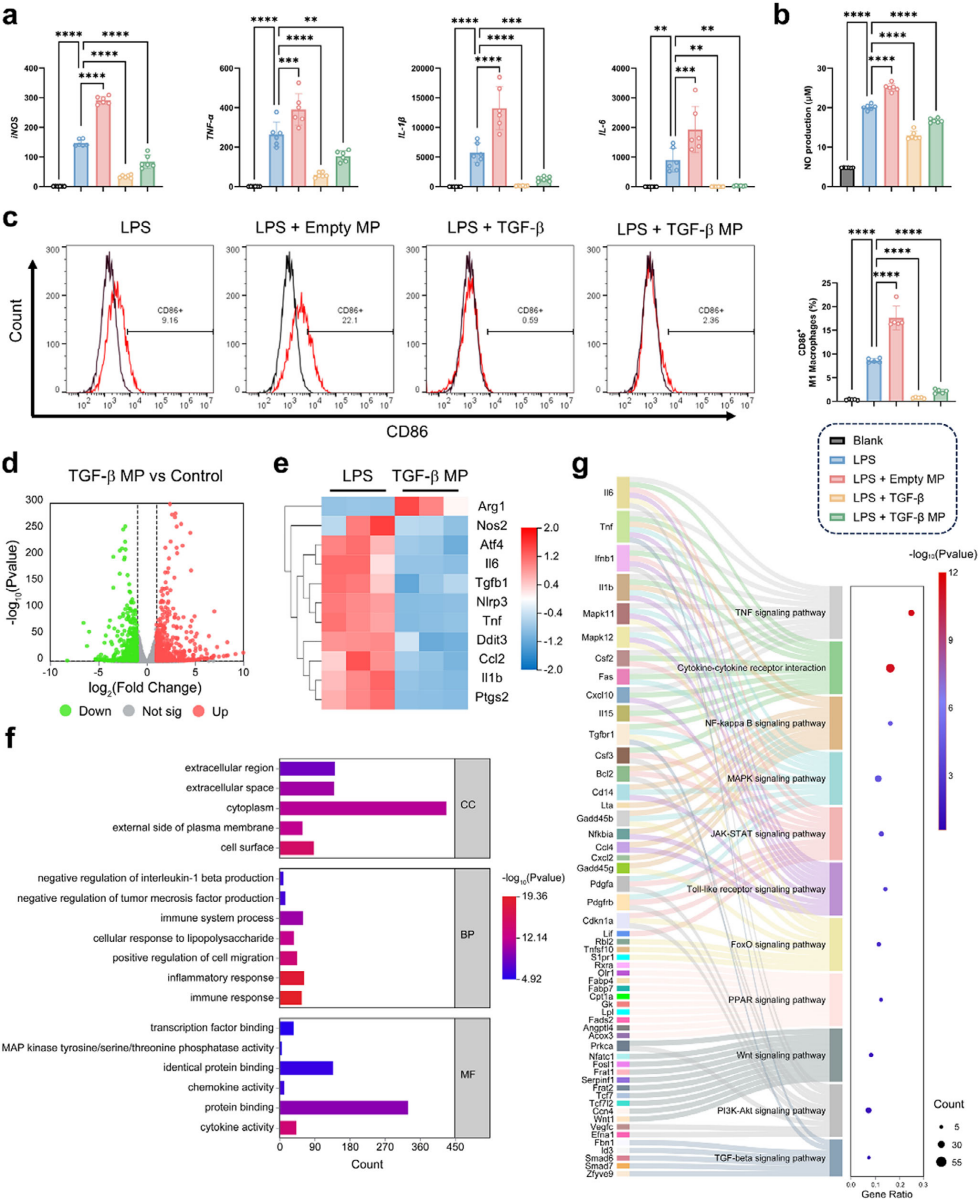
Anti‐inflammatory effects of TGF‐β released from PVA‐Tyr MP MN on RAW 264.7 in vitro. (a) qRT‐PCR analysis of the expression levels of iNOS, TNF‐α, IL‐1β, and IL‐6 (*n* = 6); (b) NO production level measured with classical Griess reagent (*n* = 6); (c) flow cytometric detection of CD86^+^ cells and statistical analysis (*n* = 5). RNA‐seq analysis of LPS and TGF‐β MP groups: (d) volcano plot displaying differentially expressed genes between the LPS and TGF‐β groups (|Log2Fold Change| ≥ 1, *p*‐value < 0.05); (e) heat map of typical differential genes; (f) GO pathway‐enrichment analysis of differential genes; (g) KEGG pathway‐enrichment analysis of differential genes (*n* = 3).

As shown in Figure 2a, LPS treatment significantly increased mRNA expression level of M1 surface marker (iNOS, 147.20 folds), along with classic pro‐inflammation cytokines TNF‐α (264.40 folds), IL‐1β (5748.00 folds), and IL‐6 (899.40 folds). Interestingly, the addition of empty MP MN lysates also amplified the inflammatory response (iNOS, 291.50 folds; TNF‐α, 390.40 folds; IL‐1β, 13250.00 folds; IL‐6, 1928.00 folds). Such response is not uncommon in PVA‐based systems [58, 59]. Importantly, this effect was substantially reversed by TGF‐β incorporation. Treatment with TGF‐β MP MN lysates significantly suppressed inflammatory gene expression (iNOS, 83.78 folds; TNF‐α, 154.10 folds; IL‐1β, 1299.00 folds; IL‐6, 28.12 folds).

NO production another hallmark of macrophage‐mediated inflammation was also evaluated (Figure 2b) [60]. Elevated NO levels were observed following LPS stimulation consistent with enhanced nitrosative stress and inflammatory activity [61, 62]. Both TGF‐β MP MN lysates and free TGF‐β treatment markedly suppressed NO generation, consistent with the down regulation of inflammatory cytokines.

Flow cytometric analysis also revealed that percentage of CD86^+^ (M1 surface marker) macrophages was increased from 0.36% to 8.62% after stimulated by LPS, and addition of empty MP MN lysates further raised the positive rate to 17.62%. Conversely, treatment with TGF‐β MP MN lysate or free TGF‐β significantly reduced the percentage of CD86^+^ cells to 2.12% and 0.76%, respectively (Figure 2c).

RNA‐sequencing (RNA‐seq) analysis was further performed to investigate the mechanism of TGF‐β MP MN lysates in LPS treated RAW 264.7. As shown in Figure S4a, a total of 1066 differentially expressed genes were identified between the TGF‐β MP MN lysate and the LPS group. Further volcano plot indicated 649 upregulated and 417 downregulated genes within TGF‐β group (Figure 2d). Heat map of typical inflammation related genes (Figure 2e) demonstrated that Arg1 was upregulated while nine other genes were downregulated (Nos2, Atf4, Il6, Tgfb1, Nlrp3, Tnf, Ddit3, Ccl2, Il1b, and ptgs2). Among these genes, Nos2 (iNOS), Il6, Tgfb1, and Tnf displayed the same expression trend in our quantitative real‐time polymerase chain reaction (qRT‐PCR) testing result (Figure 2a). Arg1 is a well‐recognized marker for M2 polarization. The rest six genes contributed to the pro‐inflammatory response. GO enrichment analysis further revealed significant enrichment of several inflammation‐related biological processes, such as Il‐1β production and tumor necrosis factor production (Figure 2f). In addition, GG pathway (Figure 2g) demonstrated that TGF‐β MP MN lysates exerted anti‐inflammation effects primarily through classical inflammation‐related TNF, NF‐kappa B, and MAPK signaling pathways with relative higher gene ratios. Furthermore, Gene Set Enrichment Analysis (GSEA) revealed significant enrichment of the ECM–receptor interaction and Adherens junction pathways (Figure S4b), supporting the anti‐inflammatory role of TGF‐β treatment. The ECM–receptor interaction pathway may modulate macrophage polarization and inflammatory signaling through cell–matrix communication, while adherens junction pathway enrichment regulates cell–cell adhesion and integrity, collectively contributing to the attenuation of LPS‐induced inflammation in RAW 264.7 cells.

### 2.3 Effects of IGF‐Loaded MP on H9c2 Under Hypoxic Environment

Cardiomyocytes apoptosis contributes significantly to adverse LV remodeling following MI, as adult cardiomyocytes exhibit limited proliferative capacity. To mitigate this, IGF‐1 were encapsulated within PVA‐Tyr MPs and then loaded into the MN. Its bioactivity was evaluated using H9c2 cells. MPs were fabricated with Ru/SPS at 0.6/6 mm to ensure sustained IGF‐1 release over a two week‐period, corresponding to the reparative and early proliferative phase.

**FIGURE 3:**
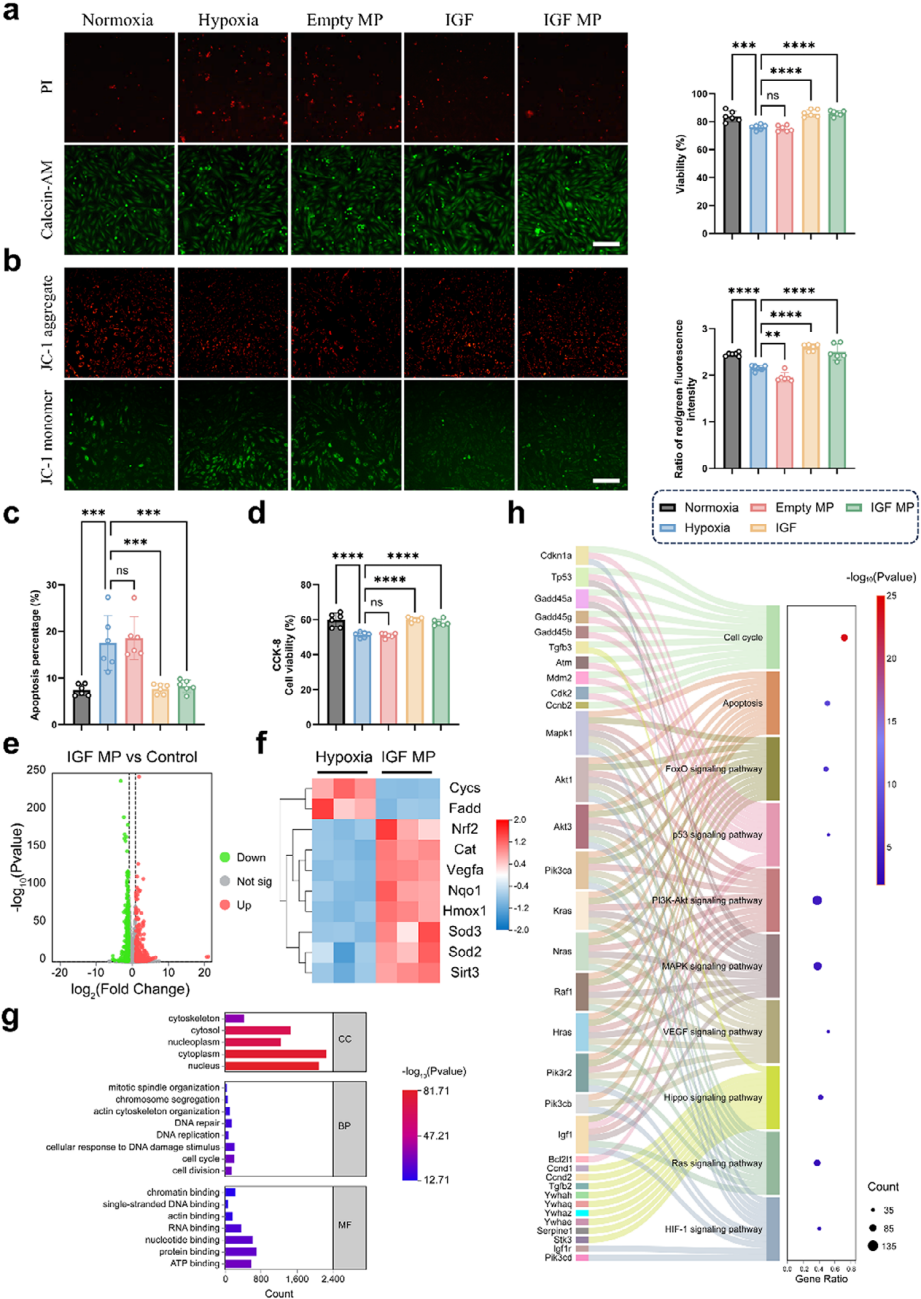
IGF‐1 released from PVA‐Tyr MP MN increased viability, reduced apoptosis rate of H9c2 in vitro. (a) Representative images of live/dead double staining and quantitative analysis; (b) representative image of JC‐1 fluorescence staining for the changes in mitochondrial membrane potential (ΔΨm) and quantitative analysis; (c) apoptosis rate determined by flow cytometry; (d) relative viability of cells measured by CCK‐8 assay (*n* = 6). Scale bar = 200 µm. RNA‐seq analysis of hypoxia and IGF MP groups: (e) volcano plot displaying differentially expressed genes between the hypoxia and IGF MP groups. (|Log2Fold Change| ≥ 1, *p*‐value < 0.05); (f) heat map of differential genes; (g) GO pathway‐enrichment analysis of differential genes; (h) KEGG pathway‐enrichment analysis of differential genes (*n* = 3).

H9c2 cells were cultured to 70%–80% confluence and then subjected to hypoxic stress (1% O_2_) for 24 h to simulate ischemic conditions. As shown in Figure 3a, live/dead cell staining (Calcein‐AM/PI) revealed extensive hypoxia‐induced cell death. However, treatment with either IGF MP MN lysates or free IGF‐1 significantly improved cell viability. In contrast, treatment with empty MP MN lysates showed no significant difference compared to the hypoxia group, indicate that PVA‐Tyr MPs themselves do not exert cytotoxic effects on H9c2 cells.

To further assess mitochondrial health, JC‐1 staining was performed to evaluate mitochondria membrane potential (MMP). In intact mitochondria, JC‐1 accumulates and forms red fluorescent J‐aggregates, whereas depolarized mitochondrial promotes JC‐1 monomer formation, emitting green fluorescence. The red/green florescence intensity ratio, quantified using Image J software, served as a proxy for mitochondrial integrity. As shown in Figure 3b, hypoxic conditions markedly reduced the red/green fluorescence ratio, indicating mitochondrial dysfunction. Notably, both IGF MP MN lysates and free IGF‐1 exhibited protective effects on the mitochondria.

Apoptosis rate and viability were also comprehensively evaluated by flow cytometric analysis of annexin V‐FITC/PI staining (Figures 3c and S5), CCK‐8 assay (Figure 3d), and TUNEL staining (Figure S6). While hypoxia treatment significantly increased apoptosis percentage and decreased viability, IGF MP MN and free IGF‐1 treatments demonstrated significant protective effects. Besides, empty MP MN lysates indicated no distinct influence in terms of the apoptosis and viability.

RNA‐seq analysis was also performed to study the mechanism of IGF MP MN lysates in H9c2 cells under hypoxic condition. A total of 896 differentially expressed genes (518 upregulated, 378 downregulated) were identified between the IGF MP and hypoxia groups (Figures 3e and S7a). Heat map of apoptosis‐related genes revealed that Cycs and Fadd were downregulated, while eight genes (Nrf2, Cat, Vegfa, Nqo1, Hmox1, SOD2, SOD3, Sirt3) were upregulated (Figure 3f). Among these genes, Cycs and Fadd are known to promote apoptosis, whereas the other eight genes exert anti‐apoptosis effects. GO analysis and KEGG signaling pathways were presented in Figure 3g and Figure 3h, respectively. GO analysis revealed significant enrichment in apoptosis and cell cycle‐related processes (e.g., DNA repair, DNA replication, and cellular response to DNA damage stimulus) (Figure 3g). KEGG pathway analysis (Figure 3h) demonstrated that IGF MP MN lysates treatment contributed to high enrichment of genes in cell cycle‐ and apoptosis‐related pathways. FoxO, P53, and PI3K/Akt signaling pathways (classical cell cycle and apoptosis related pathways) were confirmed as key pathways contributing to the anti‐apoptosis capacity of IGF MP MN lysates treatment. Furthermore, GSEA provided additional depth to these findings by revealing coordinated activation of broader functional programs (Figure S7b). Specifically, the significant enrichment of the cardiac muscle contraction pathway underscores the treatment's efficacy in not only promoting cell survival but also in potentially preserving the core function of H9c2 under hypoxia. Concurrently, the enrichment of the ABC transporters pathway highlights a reinforced cellular defense mechanism. The upregulation of this pathway, which is involved in the efflux of toxins and the maintenance of metabolic homeostasis, likely works in concert with the observed antioxidant and anti‐apoptotic gene expression (e.g., Nrf2, SOD2, Hmox1) to protect the cells from hypoxic damage [63, 64].

### 2.4 Effects of VEGF‐Loaded MP on Endothelial Cells

**FIGURE 4:**
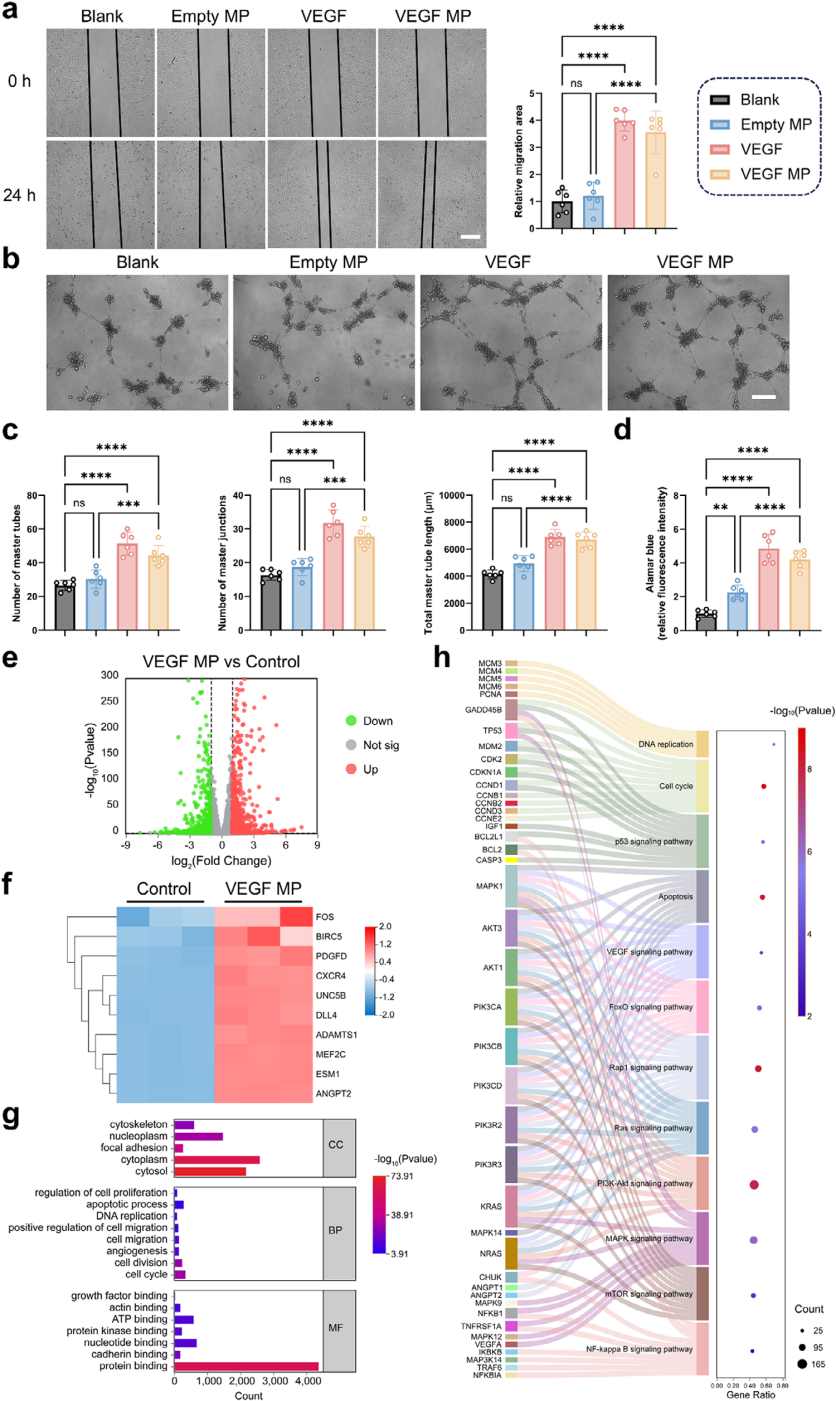
Effects of VEGF released from PVA‐Tyr MP MN on HUVEC (a) migration and quantification, (b) tube formation and (c) quantification, and (d) proliferation profile (*n* = 6). RNA‐seq analysis of control and VEGF MP groups: (e) volcano plot displaying differentially expressed genes between the control and VEGF MP groups. (|Log2Fold Change| ≥ 1, *p*‐value < 0.05); (f) heat map of differentially expressed genes; (g) GO pathway‐enrichment analysis of differential genes; (h) KEGG pathway‐enrichment analysis of differential genes (*n* = 3). Scale bar = 200 µm (a,b).

Angiogenesis plays a pivotal role in post‐infarction cardiac repair by restoring microvascular perfusion, preserving cardiomyocytes, and facilitating functional recovery. To harness this effect, VEGF was encapsulated into MPs and then loaded into MN. Bioactivity of the released VEGF was evaluated in human umbilical vein endothelial cells (HUVECs). MPs were fabricated using Ru/SPS at a concentration of 0.8/8 mm to achieve sustained VEGF release over a four‐week period. To assess the effects of VEGF MP MN lysates on migration ability of HUVECs, a wound healing assay was performed. As depicted in Figure 4a, both VEGF MP MN lysates and free VEGF significantly promoted HUVEC migration into wound area, increasing the covered area approximately fourfold after 24 h relative to the control. In contrast, empty MP MN lysates had no significant effect on cell migration.

To evaluate the proangiogenic effects of released VEGF, HUVECs were seeded on Matrigel‐coated wells (GF reduced) and incubated with VEGF MP MN lysates for 12 h. As shown in Figure 4b,c, both VEGF MP MN lysates and free VEGF significantly enhanced tube formation, as indicated by increased master tube numbers, master junction counts, and total tube length, compared to the control and empty MP MN lysate groups. The empty MP MN lysate group showed a slight increase in junction number. Its proangiogenic effect was negligible.

HUVEC proliferation in response to VEGF release was assessed using the Alamar Blue assay. As shown in Figure 4d, treatment with either VEGF MP MN lysates or free VEGF resulted in over a threefold increase in fluorescence intensity compared to the control group, indicating enhanced cellular proliferation. Interestingly, empty MP MN lysates also promoted proliferation to certain degree. This may be attributed to the presence of the tyramine moiety, which remains intact following ester bond hydrolysis of the PVA‐Tyr hydrogel. This finding aligns with previous reports demonstrating that tyramine‐containing polymers support the proliferation of mammalian cells, including L929 fibroblasts and human embryonic kidney cells [44, 65].

RNA‐seq analysis was conducted to investigate the mechanism of VEGF MP MN lysates in HUVECs. There were 1124 differentially expressed genes between the control and VEGF MP MN groups, comprising 614 upregulated and 510 downregulated genes (Figures 4e and S8a). Heat map of pro‐angiogenetic, pro‐proliferative, and anti‐apoptopic genes displayed upregulated trend (FOS, BIRC5, PDGFD, CXCR4, UNC5B, DLL4, ADAMTS1, MEF2C, ESM1, ANGPT2). Further GO analysis and KEGG signaling pathways were presented in Figure 4g and Figure 4h, respectively. GO revealed significant enrichment in proliferation, apoptosis, migration, and angiogenesis‐related processes (Figure 4g). KEGG pathway analysis (Figure 4h) demonstrated enrichment of genes in classical apoptotis‐ and angiogenesis‐related pathways (P53, VEGF, FoxO, Rap1, Ras, PI3K‐AKT, MAPK, mTOR, and NF‐kappaB signaling pathways). Furthermore, GSEA (Figure S8b) corroborated the promotive effect of VEGF MP treatment on HUVECs, revealing significant enrichment in the cell cycle and DNA replication pathways. The enrichment of these pathways indicates that VEGF MP MN lysate treatment potently enhances the proliferative capacity of HUVECs. This provides further mechanistic support for its positive role in angiogenesis.

Additional supportive in vitro validation of the combined GF/GF‐MP formulation across multiple relevant cell types is provided in the Supporting Information (Figures S9–S11).

### 2.5 The Cardioprotection Potential of MN Patch

**FIGURE 5:**
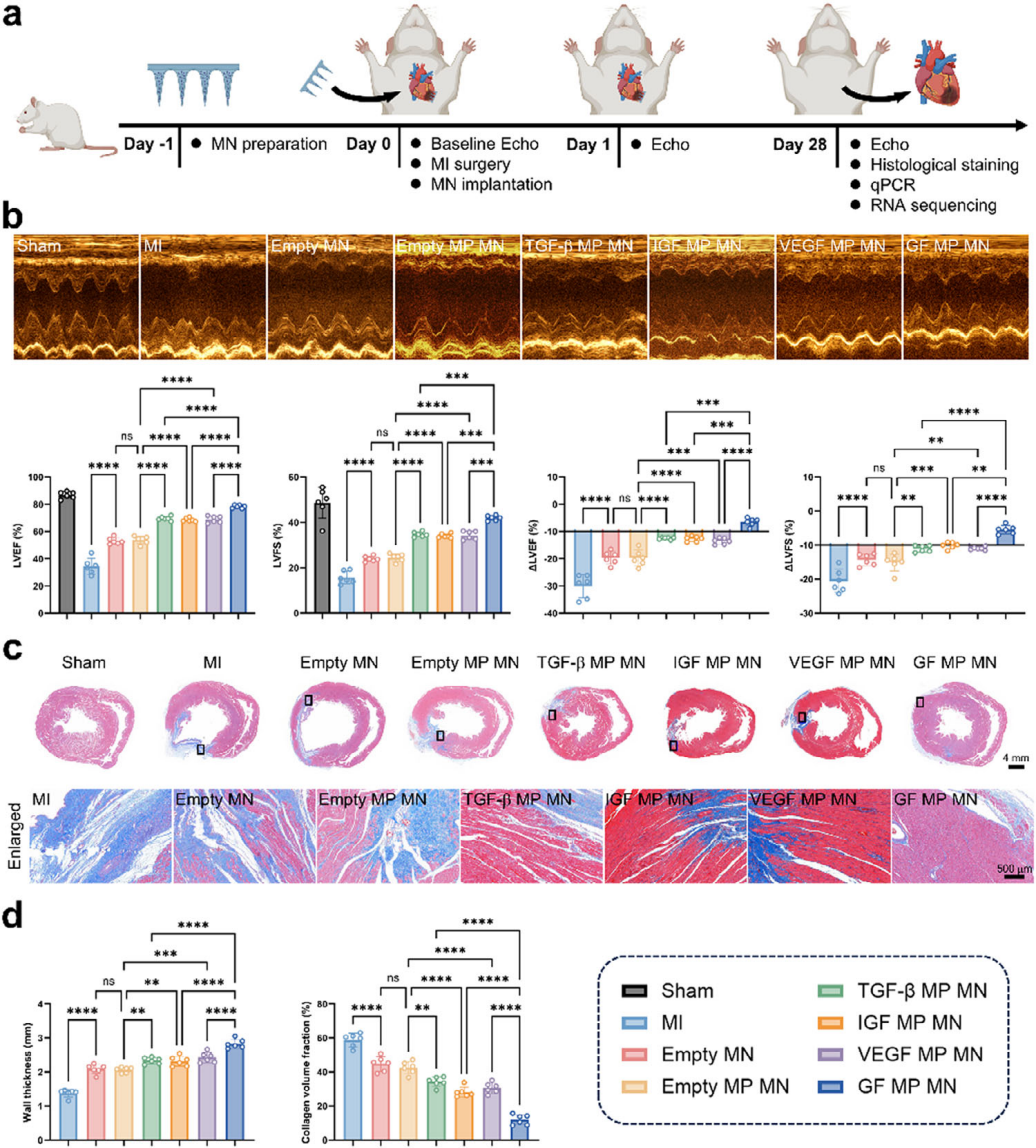
Therapeutic effects of GF MP MNs on the rat model of MI. (a) Experimental scheme of the MI model construction and MN treatment in rats; (b) representative images of M‐mode echocardiography at day 28 post MI, and quantification of LVEF, LVFS, ΔLVEF, and ΔLVFS (*n* = 6); (c) Masson trichrome staining of infracted sites after different treatments for 4 weeks and (d) quantification of wall thickness and collagen deposition area (*n* = 6). Panel (a) was partially created in BioRender. Cui, S. (2026) https://BioRender.com/e0h1ivt.

Previous in vitro experiments have confirmed the tailorable release of the protein from PVA‐Tyr MPs loaded in MNs and preserved bioactivities of released GFs (TGF‐β, IGF‐1, VEGF). To further assess the benefits of temporal releasing multiple GFs for cardiac tissue, we used the MI model to compared therapeutic effects between of single GF deliver and multiple GFs temporal deliver (Figure 5a). As shown in Figure S12, the MN patch could be readily attached to the MI site. Moreover, Video S1 shows that the patch maintained intimate contact with the beating heart during application, further supporting the flexibility and conformability of the PVA‐based backing layer. Cardiac echocardiography was performed and quantified by left ventricular ejection fraction (LVEF) and left ventricular fractional shortening (LVFS) at day 1 and day 28 after the surgery, and ΔLVEF together with ΔLVFS were calculated. Unsurprisingly, both LVEF and LVFS of MI group were remarkably decreased in the MI group, whereas treatment with empty MN and empty MP MN partially ameliorated the condition. However, no significant therapeutic difference between empty MN and empty MP MN (Figure 5b). In contrast, single GF treatment groups (TGF‐β/IGF/VEGF MP MN) demonstrated improved cardiac function, compared with the empty MP MN group. Notably, multiple GFs treatment group (GF MP MN) demonstrated the most improved therapeutic effect in terms of the cardiac function preservation compared to all the groups. Same result was confirmed in ΔLVEF and ΔLVFS (Figure 5b).

The structural morphology (Masson and Sirius red staining, Figures 5c and S13) of the heart demonstrated that the MI group exhibited severe MI, characterized by ventricular enlargement, ventricular wall thinning, and extensive scar fibrotic tissues deposition at the infarction site (Figure 5c). By comparison, empty MN and empty MP MN groups partially mitigated these pathological changes, which TGF‐β/IGF/VEGF MP MN MP treatment groups provided further improvement (Figure 5c). The most pronounced improvement was observed in the GF MP MN treatment group. Quantification of LV wall thickness and collagen deposition (Figure 5d) results aligned with the histological findings, indicating the effectiveness of the GF MP MN treatment.

### 2.6 Multiple GFs Treatment Promoted Positive Cardiac Remodeling

**FIGURE 6:**
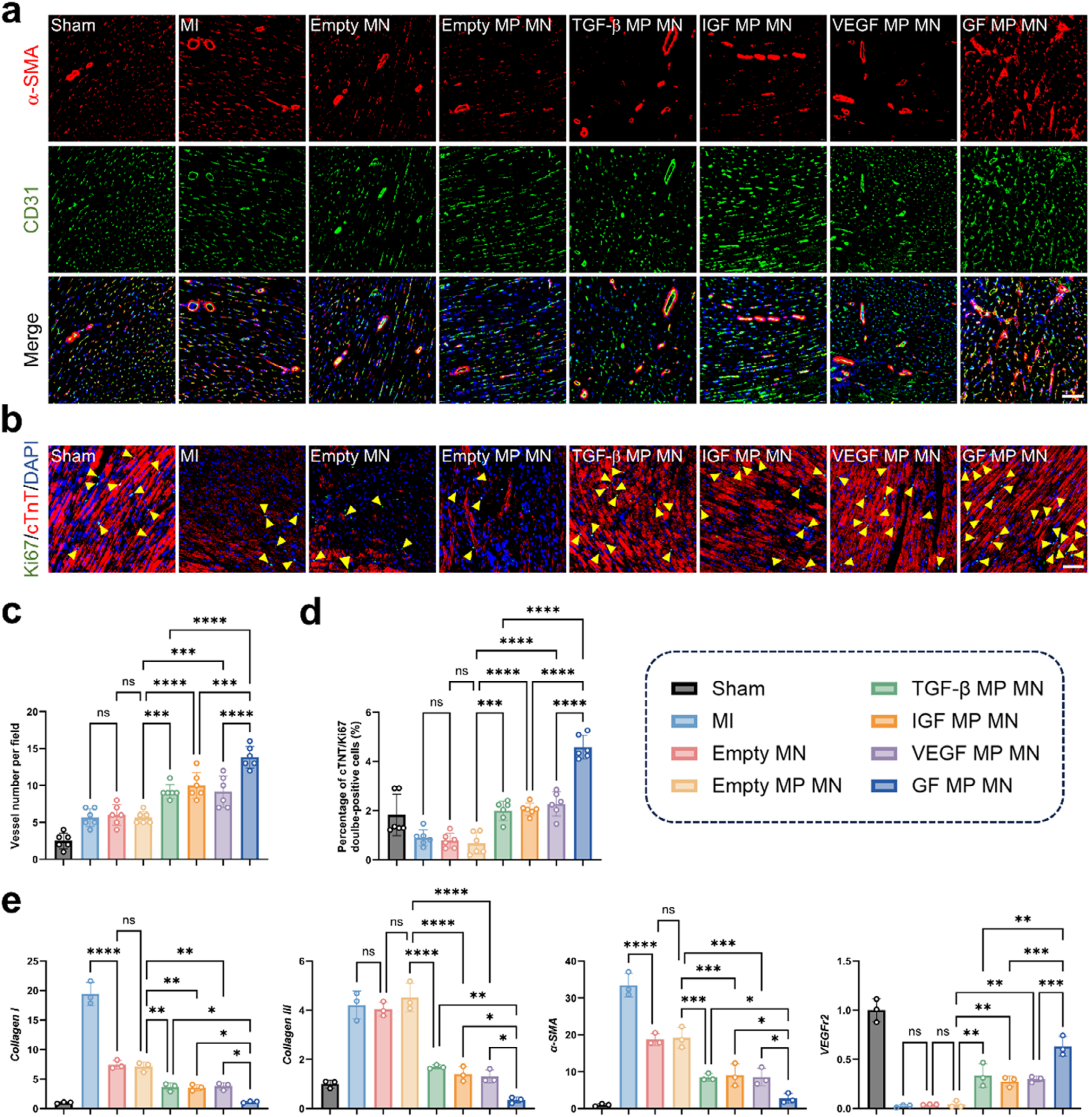
Effects of GF MP MNs on MI rat model at day 28. Immunostaining of (a) α‐SMA/CD31, (b) Ki67/cTnT at Day 28 post MI, and (c,d) quantification (*n* = 6); (e) relative mRNA expression of collagen I, collagen III, α‐SMA, and VEGFr2 from the infarction area (*n* = 3). Scale bar = 100 µm (a,b).

To further investigate the roles of GF MP MN treatment on angiogenesis and proliferation of the MI rat model, immunostaining of α‐SMA/CD31 and Ki67/cTnT were performed on the infarction area, respectively. As shown in Figure 6a–d, both the number of α‐SMA/ CD31 double positive vessels and percentage of Ki67/cTnT double positive proliferating cells demonstrated no difference among MI, empty MN, and empty MP MN groups. Treatment with TGF‐β/IGF/VEGF MP MN substantially increased the number of newly formed vessel and percentage of proliferative cells. More significantly, GF MP MN treatment further increased the vessel number and proliferating cell percentage compared with TGF‐β/IGF/VEGF MP MN groups.

Relative mRNA expression of fibrosis and vascularization‐related genes was also evaluated by qRT‐PCR (Figure 6e). Compared with MI group, treatment with empty MN significantly reduced collagen I and α‐SMA expression, while no significant changes were observed for collagen III and VEGFR2. Moreover, there were no significant differences between empty MN and empty MP MN groups across all four genes. In contrast, TGF‐β/IGF/VEGF MP MN treatment significantly increased VEGFR2 expression and decreased collagen I, collagen III, and α‐SMA expression. GF MP MN further strengthened these effects, consistent with the echocardiographic and histological results.

### 2.7 GFs Regulated Multiple Signaling Pathways for the Improved Cardiac Protection

**FIGURE 7:**
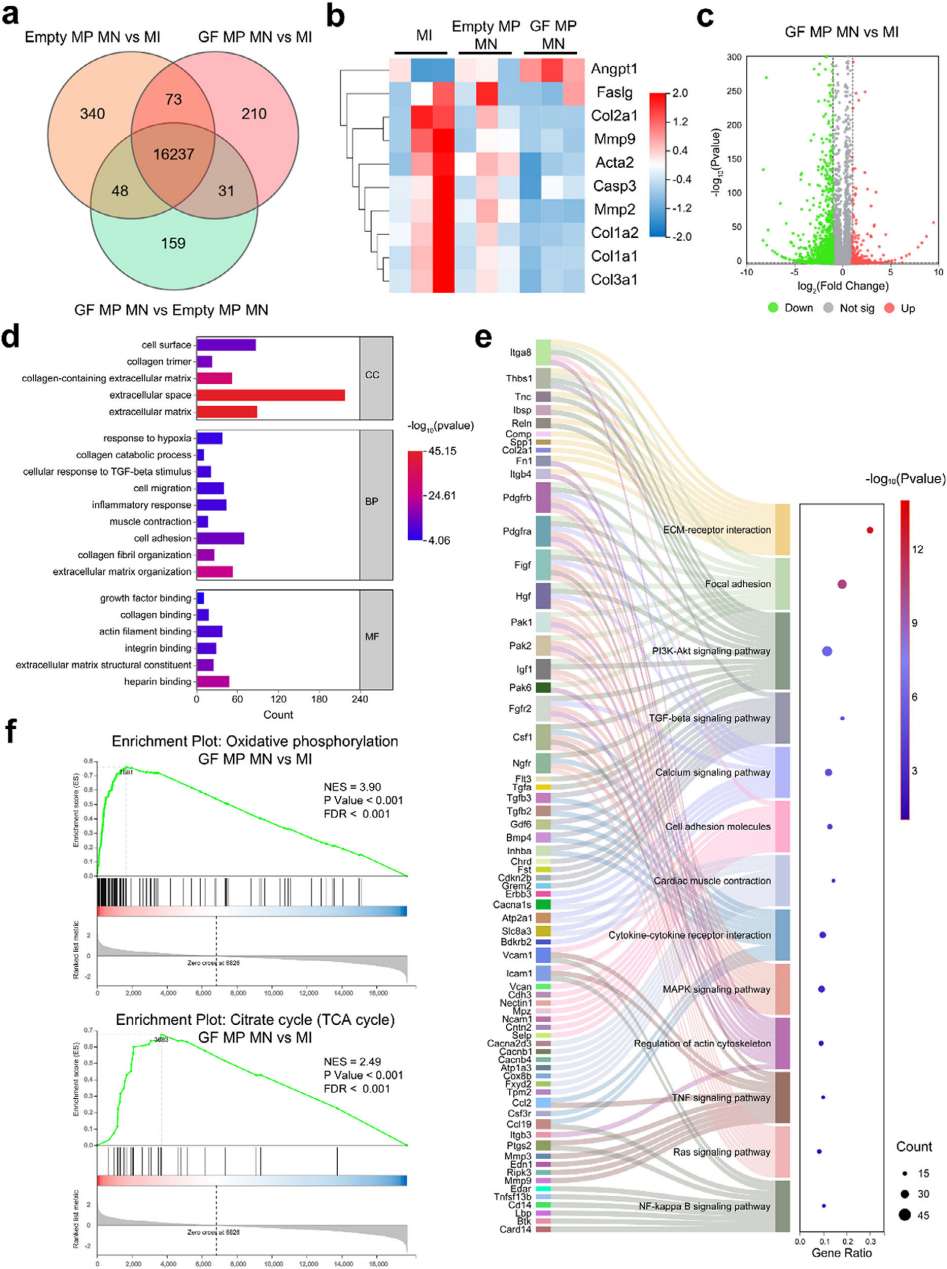
RNA‐seq analysis of MI, Empty MP MN, and GF MP MN groups at day 28: (a) Venn diagram of differentially expressed gene intersections among three groups; (b) heat map of differential genes among MI, empty MP MN, and GF MP MN groups; (c) volcano plot of differentially expressed genes between GF MP MN and MI groups (|Log2Fold Change| ≥ 1, *p*‐value < 0.05); (d) GO pathway‐enrichment analysis of differential genes between GF MP MN and MI groups; (e) KEGG pathway‐enrichment analysis of differential genes between GF MP MN and MI groups. (f) GSEA analysis of differential genes and enrichment plots of the most correlative pathways (*n* = 3).

RNA‐seq analysis was further conducted to comprehensive understand underlying work mechanism of GF MP MN in cardiac protection post MI. Venn diagram shows gene expression intersections among three groups (Empty MP MN vs MI, GF MP MN vs MI, GF MP MN vs Empty MP MN), in which 16 237 collectively expressed genes were detected (Figure 7a). As illustrated in Figure 7b, Col1a1, Col1a2, Col2a1, Col3a1, and MMP9 underwent downregulation after treated with Empty MP MN. GF MP MN induced further downregulation. Noticeably, although Acta2 and MMP2 showed no apparent downregulation after Empty MP MN treatment, significantly decreased expression level was observed in the GF MP MN group. These genes are known to play pivotal roles in the collagen deposition and fibrosis. Faslg and Casp3, which are involved in the cell apoptosis, also demonstrated reduced expression level under GF MP MN treatment. Angpt1, as a pro‐angiogenetic genes, was upregulated in Empty MP MN group. The upregulation was more significant in GF MP MN.

In addition, volcano plot and heat map revealed 1671 differentially expressed genes between GF MP MN and MI groups, including 283 upregulated and 1388 downregulated genes (Figures 7c and S14a). GO, KEGG, and GSEA analyses of these genes were further conducted to facilitate understanding of involved biological processes and pathways (Figure 7d–f). GO analysis demonstrated significant enrichment in ECM remodeling, inflammatory response, and cell adhesion/migration‐related biological processes (Figure 7d), consistent with the known activities of TGF‐β, IGF‐1, and VEGF. KEGG analysis further highlighted ECM–receptor interaction, focal adhesion, PI3K/Akt, and TGF‐β signaling pathways (Figure 7e). In addition, GSEA revealed enrichment of oxidative phosphorylation, the citrate cycle (TCA cycle), propanoate metabolism, and cardiac muscle contraction pathways (Figures 7f and S14b), collectively supporting coordinated structural and metabolic recovery after GF MP MN treatment.

To further dissect the contribution of the carrier alone, we performed a supplementary pairwise transcriptomic analysis for Empty MP MN versus MI (Figure S15). The volcano plot revealed 302 differentially expressed genes between the two groups, including 123 upregulated and 179 downregulated genes (Figure S15a). Figure S15b shows the GO enrichment results highlighting biological processes related to ECM remodeling (e.g., ECM organization) together with limited contractile recovery (e.g., cardiac muscle contraction, regulation of muscle contraction, and actin‐mediated cell contraction), whereas Figure S15c,d summarizes the corresponding GSEA and KEGG enrichment profiles. Compared with MI, Empty MP MN mainly showed enrichment of pathways related to ECM remodeling (e.g., ECM–receptor interaction and focal adhesion) together with limited contractile/metabolic recovery (e.g., oxidative phosphorylation, the citrate cycle [TCA cycle], propanoate metabolism, and cardiac muscle contraction).

To further evaluate the additional benefit conferred by temporally programmed GF delivery, we also performed a supplementary pairwise transcriptomic analysis for GF MP MN versus Empty MP MN (Figure S16). The volcano plot revealed 462 differentially expressed genes between the two groups, including 136 upregulated and 326 downregulated genes (Figure S16a). Figure S16b presents the GO enrichment results, which further emphasize biological processes associated with ECM remodeling and cell‐adhesion/migration‐related repair (e.g., ECM organization, collagen fibril organization, cell adhesion, cell‐matrix adhesion, and cell migration), while Figure S16c,d illustrates the corresponding GSEA and KEGG enrichment results. Compared with Empty MP MN alone, GF MP MN further highlighted ECM‐/adhesion‐associated and reparative signaling programs (e.g., ECM–receptor interaction, focal adhesion, PI3K/Akt signaling, and TGF‐β signaling) together with metabolic recovery programs (e.g., oxidative phosphorylation, the citrate cycle [TCA cycle], propanoate metabolism, and cardiac muscle contraction), supporting that GF loading reinforced reparative remodeling beyond the empty carrier alone.

### 2.8 Anti‐inflammatory and Anti‐apoptotic Effects of the GF MN Patch During Inflammatory Phase

**FIGURE 8:**
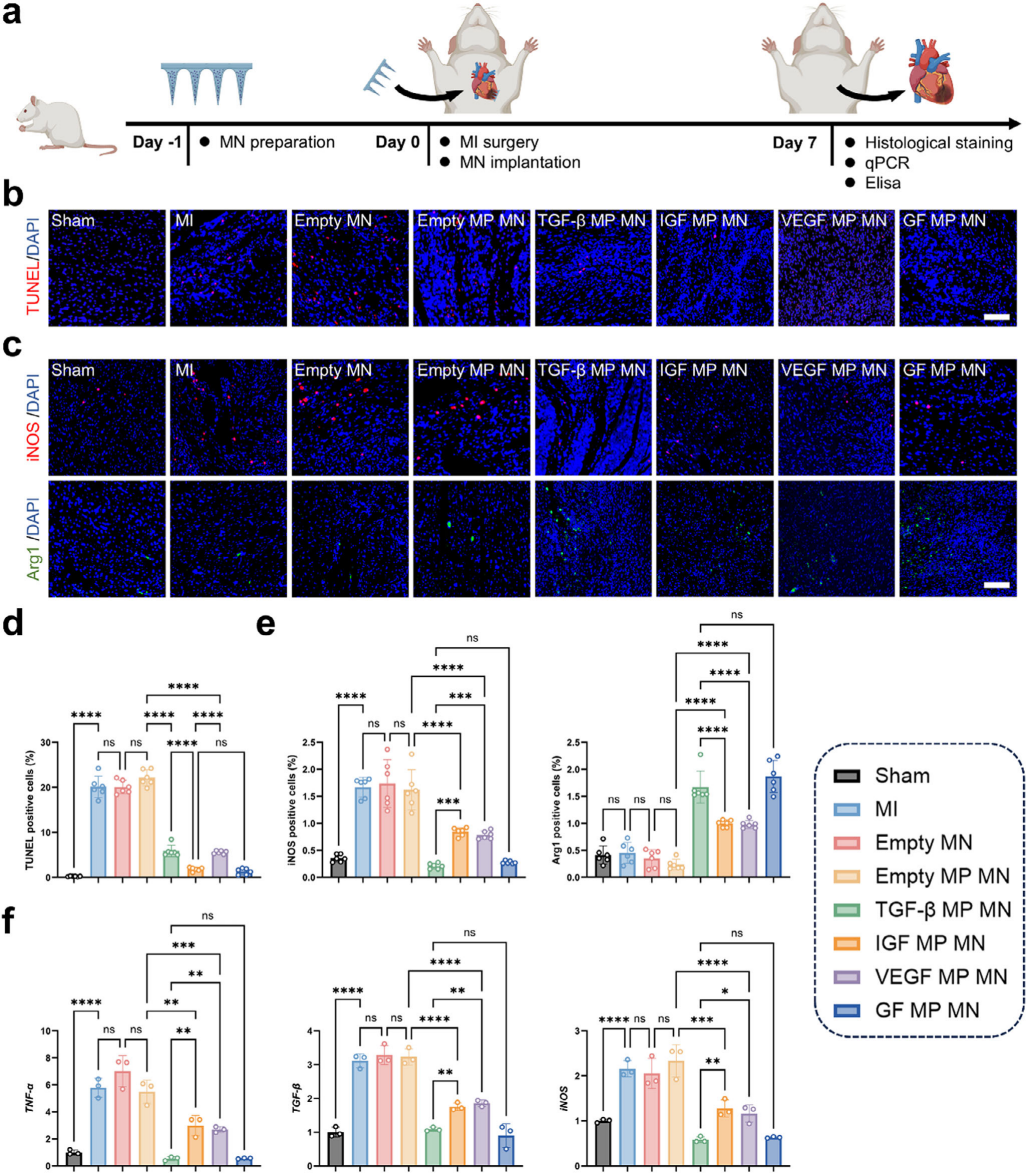
Effects of GF MP MNs on inflammation and apoptosis at day 7. (a) Schematic diagram of the MI model construction and experimental arrangements for the evaluation of inflammation and apoptosis in rats. Representative (b) TUNEL staining, (c) iNOS, and Arg1 staining of the myocardium, and (d,e) quantification (*n* = 6); (f) relative mRNA expression of TNF‐α, TGF‐β, and iNOS (*n* = 3). Panel (a) was partially created in BioRender. Cui, S. (2026) https://BioRender.com/n9hwll5.

Given that previous results have confirmed the cardio‐protection function of GF MN treatment at day 28 post MI, we further conducted experiments to verify the anti‐inflammatory and anti‐apoptotic effects at day 7. Schematic diagram was illustrated in Figure 8a, which outlines the critical time points and experimental arrangements. In TUNEL staining, the MI group showed a significantly increased apoptotic rate compared with the sham group, whereas Empty MN group and Empty MP MN group showed no significant difference versus MI. In contrast, both TGF‐β MP MN group and VEGF MP MN group significantly reduced TUNEL positivity. IGF‐1 MP MN group further reduced the apoptotic rate relative to the TGF‐β MP MN and VEGF MP MN groups, with no significant difference compared with the GF MP MN group (Figure 8b,d).

For inflammatory marker staining, the MI group showed a markedly increased iNOS‐positive rate compared with the sham group, while Empty MN and Empty MP MN again showed no significant difference versus MI. Both IGF‐1 MP MN and VEGF MP MN significantly reduced iNOS positivity, whereas TGF‐β MP MN group further reduced the iNOS‐positive rate. TGF‐β MP MN group showed no significant difference compared with the GF MP MN group. In addition, Arg1 positivity showed no obvious difference among the sham, MI, Empty MN, and Empty MP MN groups. It was significantly increased by IGF‐1 MP MN and VEGF MP MN treatment, and further increased in the TGF‐β MP MN group. No significant difference was observed between the TGF‐β MP MN group and GF MP MN group (Figure 8c,e).

Consistent with the staining results, myocardial qRT‐PCR analysis at day 7 showed that TNF‐α, TGF‐β, and iNOS expression levels were significantly increased in the MI group compared with the sham group, whereas Empty MN and Empty MP MN again showed no significant difference versus MI (Figure 8f). Both IGF‐1 MP MN and VEGF MP MN significantly reduced the expression of these genes compared with the MI, Empty MN, and Empty MP MN groups. Notably, TGF‐β MP MN further reduced TNF‐α, TGF‐β, and iNOS expression relative to the IGF‐1 MP MN and VEGF MP MN groups, with no significant difference compared with the GF MP MN group. Taken together, these results indicate that the single‐factor treatment groups exhibited distinct early in vivo effects on apoptosis and inflammatory regulation.

Elisa assay was also conducted to further validate the anti‐inflammatory effects of GF MP MN treatments. As shown in Figure S17, serum levels of TNF‐α, IL‐1β, and MCP‐1 exhibited no significant changes among MI, Empty MN, and Empty MP MN groups, while GF MP MN significantly reduced their concentrations.

**FIGURE 9:**
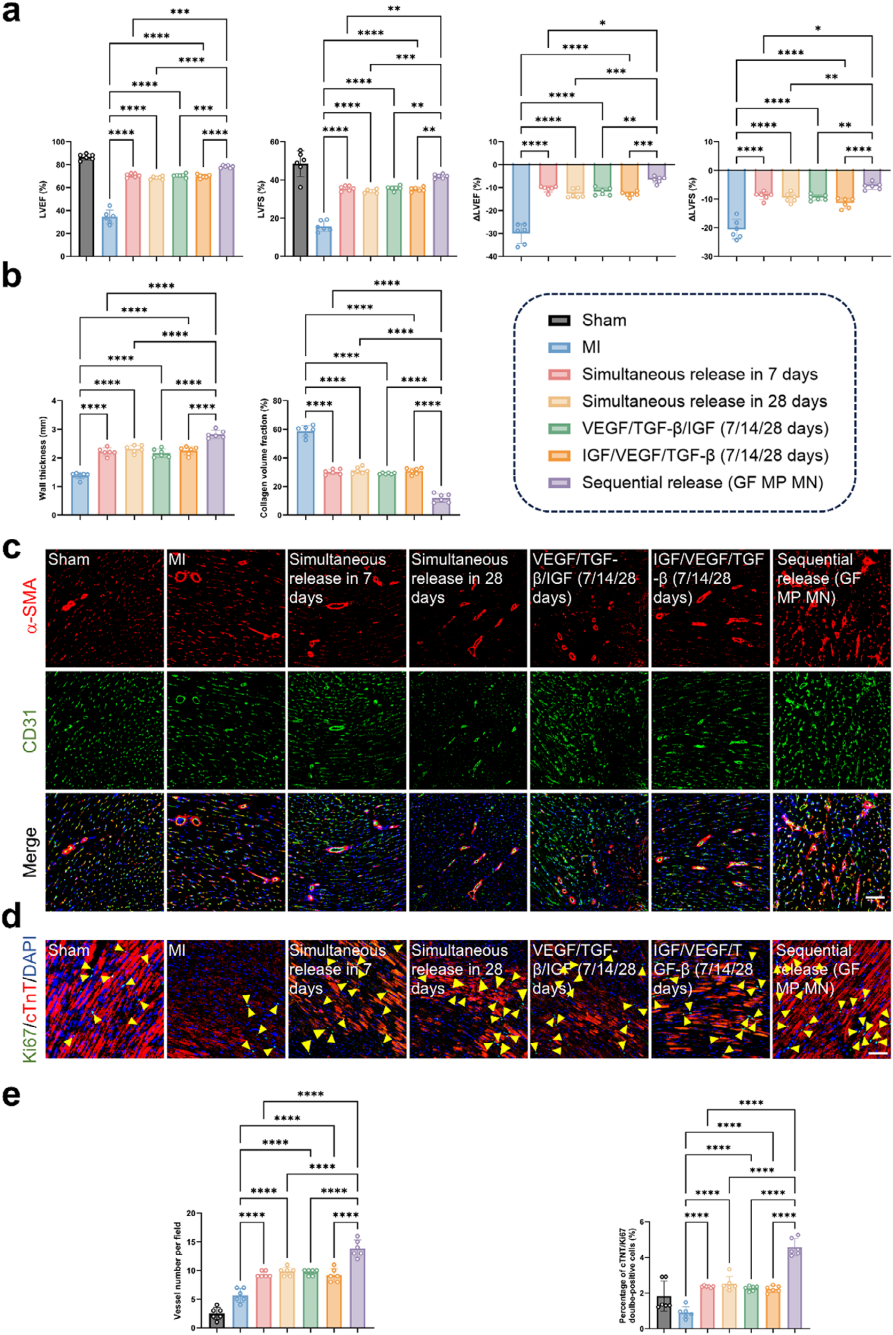
Direct in vivo comparison of triple‐GF release schedules at day 28 after MI. (a) LVEF, LVFS, ΔLVEF, and ΔLVFS; (b) infarct‐wall thickness and collagen volume fraction; (c) α‐SMA (red)/CD31 (green) co‐immunofluorescence; (d) cTnT (red)/Ki67 (green) co‐immunofluorescence; (e) vessel number per field and percentage of cTnT/Ki67 double‐positive cells (*n* = 6). Nuclei were counterstained with DAPI (blue), and arrowheads in (d) indicate double‐positive cells. Scale bar = 100 µm (c,d). The tested schedules included simultaneous release within 7 or 28 days, VEGF/TGF‐β/IGF‐1 or IGF‐1/VEGF/TGF‐β release within 7/14/28 days, and sequential TGF‐β/IGF‐1/VEGF release within 7/14/28 days (original GF MP MN group).

The newly added in vivo schedule‐control study further distinguished kinetic staggering from simple duration extension. The original TGF‐β/IGF‐1/VEGF program outperformed both 7‐ and 28‐day simultaneous‐window delivery, as well as two order‐swapped 7/14/28‐day programs, across functional, structural, angiogenic, and cardiomyocyte‐proliferation readouts (Figures 9, S18, and S19).

### 2.9 Direct In Vivo Comparison of Alternative Triple‐GF Release Schedules

To determine whether therapeutic efficacy depended on the temporal order of GF release, rather than on triple‐GF co‐delivery alone, we performed an additional in vivo schedule‐control study using four alternative triple‐GF MP‐in‐MN formulations. These included two simultaneous‐release schedules, in which all three GFs completed release within either 7 or 28 days, and two order‐swapped staggered schedules, in which VEGF/TGF‐β/IGF‐1 or IGF‐1/VEGF/TGF‐β were assigned to 7/14/28‐day release windows. The original TGF‐β/IGF‐1/VEGF 7/14/28‐day program was used as the phase‐matched sequential comparator.

At day 28, all four alternative schedules significantly improved LVEF, LVFS, ΔLVEF, and ΔLVFS compared with the MI group, indicating that each triple‐GF delivery program provided functional benefit. However, each alternative schedule remained significantly less effective than the original TGF‐β/IGF‐1/VEGF sequential program (Figures 9a and S18). Consistent results were observed in the histological analyses. All four alternative schedules increased infarct‐wall thickness and reduced collagen volume fraction relative to MI (Figures 9b and S19), increased α‐SMA/CD31‐positive vessel number (Figure 9c,e), and increased the percentage of cTnT/Ki67 double‐positive cells (Figure 9d,e). Nevertheless, for each of these endpoints, the original sequential program produced significantly greater improvement than any of the alternative schedules. The cTnT/Ki67 result was interpreted as evidence of cardiomyocyte cell‐cycle activity, rather than definitive proof of completed cytokinesis.

These direct schedule‐control experiments demonstrate that the therapeutic benefit of GF MP MNs cannot be attributed solely to the presence of three GFs, nor simply to extending the release duration of all three factors. If triple‐GF co‐delivery alone were sufficient, the five triple‐GF groups would be expected to show comparable efficacy. Likewise, if prolonged release were the dominant determinant, the 28‐day simultaneous‐release group would be expected to approach the original sequential group. Instead, the TGF‐β/IGF‐1/VEGF 7/14/28‐day program consistently outperformed both simultaneous‐release schedules and both order‐swapped staggered schedules across functional, structural, angiogenic, and cardiomyocyte cell‐cycle readouts. These findings support the importance of phase‐matched temporal allocation for coordinated cardiac repair under the tested formulation conditions.

## 3 Discussion

Effective cardiac regeneration depends on temporal coordination of multiple repair cues. In this work, the PM‐MN platform uses tunable PVA‐Tyr MP degradation to program a phase‐biased and kinetically staggered release of TGF‐β, IGF‐1, and VEGF, three essential mediators of myocardial repair. Rather than simply prolonging factor retention, this design encodes therapeutic timing directly into the material, providing a programmable acellular strategy for coordinated cardiac repair.

The present study establishes a programmable PM‐MN system that reproduces a phase‐biased and kinetically staggered release of TGF‐β, IGF‐1, and VEGF, three essential mediators of myocardial repair.

In biological systems, the timing of molecular cues often dictates tissue outcomes as much as their identity or concentration. During cardiac repair using the stem cell therapy, stem cells secrete different GFs in a phase‐dependent manner, collectively guiding inflammation, matrix remodeling, and angiogenesis [12, 13, 14, 15, 16, 17, 18, 19, 54, 66, 67, 68]. Replicating this dynamic temporal sequence using synthetic materials has been a long‐standing challenge. Here, we demonstrate that by manipulating the Ru/SPS photo‐initiated crosslinking density in the PVA‐Tyr macromer, degradation and release kinetics can be systematically tuned, generating phase‐biased, kinetically staggered, and partially overlapping GF release over a period of weeks. Notably, although MP diameter varied across Ru/SPS conditions, the particle‐size trend did not explain the release trend. The 0.4/4 mm group showed the largest MPs, yet released its cargo most rapidly, whereas higher Ru/SPS groups produced smaller MPs but exhibited slower degradation and more prolonged release. Thus, while MP size may contribute to the release profile to some extent, particularly during the initial diffusion‐driven burst phase, it is unlikely to be the primary parameter controlling the overall release kinetics in this system. This interpretation is consistent with previous study on PVA‐Tyr hydrogels, which showed that increasing crosslinking density prolonged hydrogel degradation and correspondingly extended the release time of incorporated proteins/GFs, with release duration matching the degradation timeframe [45]. This controlled hydrolysis‐driven release differs from diffusion‐ or erosion‐dominated mechanisms in traditional hydrogels, where release profiles are largely passive and uncontrollable.

In our system, three GFs shared an initial burst release but subsequently diverged into distinct dominant windows, with TGF‐β, IGF‐1, and VEGF completing release at approximately 5, 15, and 29 days, respectively. This design was intended to mimic the staged paracrine secretion logic of stem cell therapy after MI rather than to maximize the persistence of all factors equally. The amount of GF released during the burst phase could, in principle, be tuned by adjusting the loading concentration of each factor, which may help mitigate potential adverse effects associated with rapid early GF release. Furthermore, TGF‐β was intentionally assigned to the earliest dominant window because its effects after MI are strongly time‐dependent: early TGF‐β activity supports inflammatory resolution and infarct healing, whereas sustained signaling is associated with fibrosis and adverse remodeling [69, 70, 71, 72]. IGF‐1 was positioned as an intermediate‐duration cue to span the reparative and early proliferative phase, during which cardiomyocyte survival remains important. Prior studies support that IGF‐1 should not be limited to very short‐lived exposure, while prolonged or excessive IGF‐1 signaling may also engage trophic and pro‐remodeling pathways [73, 74, 75, 76, 77]. By contrast, VEGF was intentionally assigned the longest dominant window, because angiogenesis requires not only endothelial sprouting but also sufficiently prolonged stimulation for vessel stabilization [78, 79, 80].

The resulting release program supported coordinated therapeutic benefits in vivo. GF MP MN treatment improved ventricular function, attenuated fibrosis, and promoted angiogenesis, while transcriptomic analyses indicated concurrent regulation of ECM remodeling, cell‐adhesion signaling, and metabolic pathways. Supplementary pairwise transcriptomic analyses further refined this interpretation by showing that the empty carrier alone was associated mainly with partial matrix/structural and metabolic adjustments, whereas GF loading further strengthened ECM‐remodeling, adhesion‐associated, and reparative signaling programs relative to Empty MP MN (Figures S15 and S16). Together, these findings show that temporally programmed GF delivery supports both structural repair and metabolic recovery in the infarcted heart, highlighting the value of time‐encoded biomaterials for myocardial regeneration. In direct schedule‐control experiments, the phase‐matched TGF‐β/IGF‐1/VEGF program produced significantly greater recovery of ventricular function, infarct‐wall preservation, reduction of collagen deposition, vascularization, and cardiomyocyte cell‐cycle activity than either simultaneous‐window delivery or two order‐swapped schedules. These findings establish temporal order as a meaningful design variable in the tested PM‐MN system, beyond the effects of triple‐factor combination or release duration alone.

The success of the PM‐MN system stems from the intimate coupling of material chemistry and biological signaling. The bi‐phenol crosslinking reaction between the tyramine groups and Ru/SPS‐generated radicals formed covalent bonds with both the polymer backbone and tyrosine residues on the GFs [44]. This covalent immobilization stabilized the proteins against enzymatic degradation and prevented premature diffusion while preserving their bioactivity. As hydrolysis progressed, ester bond cleavage gradually released bioactive factors in a predictable, stage‐specific sequence. The crosslinking density, and therefore degradation kinetics, could be continuously tuned by altering initiator concentration—a simple yet powerful lever for designing time‐controlled therapeutic systems. In the present system, this design enabled sustained release for up to ∼60 days. Notably, although sustained release was observed for up to two months, long‐term activity is expected to depend strongly on the specific recombinant construct, conjugation chemistry, and immobilization mode [81, 82, 83]. Accordingly, while this platform may offer potential for chronic indications requiring extended GF delivery, long‐term bioactivity of each candidate GF would need to be evaluated and screened according to the specific therapeutic context.

The MN configuration provided additional advantages by enabling minimally invasive, localized delivery directly to the infarcted myocardium. With a mechanical strength above 8 N (Figure S1b), the MN patch effectively penetrated the epicardial layer without causing additional tissue injury, ensuring efficient drug transfer while minimizing systemic exposure. The combination of mechanical precision, chemical programmability, and biological efficacy underscores the versatility of the PM‐MN platform.

Most biomaterial‐based cardiac delivery systems focus on sustained or triggered release, relying on diffusion gradients, external stimuli, or responsive linkers [32, 37, 38]. While these methods prolong bioavailability, they rarely reproduce the temporal logic of natural repair processes. Layer‐by‐layer hydrogels or multi‐reservoir devices can achieve stepwise release, but their complexity and limited control over overlapping kinetics constrain translational potential. By contrast, our system achieves a continuous and adjustable release sequence using a single material formulation, requiring no mechanical assembly or external triggers. This simplicity enhances reproducibility and scalability, two key prerequisites for clinical translation.

Moreover, unlike cell‐based therapies, the PM‐MN system circumvents issues of cell survival, immune rejection, and tumorigenic risk while preserving the multi‐factorial and dynamic benefits of stem cell paracrine signaling. Thus, it bridges the gap between biological complexity and material controllability—combining the richness of cellular cues with the precision of synthetic design.

The therapeutic outcomes observed in the rat MI model demonstrate the potential of temporally programmed delivery as a clinically relevant strategy. The pronounced improvements in cardiac function, reduced fibrosis, and enhanced neovascularization suggest that time‐encoded release can drive the heart toward constructive remodeling rather than scarring. Future studies could further optimize the relative dosing once factor‐specific loading and retention are quantified directly for each GF, as well as additional factorial optimization of multi‐factor stoichiometry independent of temporal programming.

Importantly, the design principles of this system are disease‐agnostic. By substituting different bioactive molecules, the PM‐MN platform could be adapted to orchestrate complex healing processes in other tissues, such as chronic wounds, ischemic limbs, or neural injuries, where temporal coordination of cytokine signaling is equally critical. The modularity of the PVA‐Tyr chemistry also facilitates adaptation to various delivery modalities, including injectable MP suspensions or patch‐based systems for minimally invasive administration. As manufacturing techniques advance, such programmable biomaterials could be integrated into next‐generation bioelectronic interfaces or smart implantables capable of responding dynamically to physiological cues. This convergence of materials engineering and temporal biology opens new directions for precision regenerative medicine.

We also acknowledge several limitations of present study. First, direct real‐time tracking of native, unlabeled GF release from the implanted PM‐MN patch in vivo remains technically challenging. Current approaches usually rely on labeled proteins or imaging‐enabled materials and therefore cannot directly measure native local release kinetics [84, 85]. Thus, direct in vivo monitoring of GF release remains a limitation of this study and a goal for future work. Second, although the additional schedule controls strengthen the temporal interpretation, formulation‐dependent retention means that the effective retained dose was not fully dose‐matched across all schedules and was estimated from formulation‐level retention rather than directly quantified for each native GF. Therefore, the present data establish the superiority of the tested TGF‐β/IGF‐1/VEGF program under the tested formulation conditions, but do not prove that the 200 ng dose or the exact release windows are globally optimal, nor do they fully separate timing from dose. Future studies should directly quantify GF‐specific retained/releasable amounts and use dose‐normalized factorial designs.

## 4 Conclusion

In summary, Ru/SPS‐dependent PVA‐Tyr crosslinking generated phase‐biased, kinetically staggered, and partially overlapping availability of TGF‐β, IGF‐1, and VEGF. Among the five tested triple‐GF schedules, the TGF‐β/IGF‐1/VEGF program outperformed both simultaneous‐window schedules and two order‐swapped programs across functional, structural, angiogenic, and cardiomyocyte‐proliferation readouts. These findings support phase‐matched temporal allocation as a meaningful design strategy for coordinated cardiac repair, while further dose‐normalized optimization will be required to establish the globally optimal program.

## 5 Experimental Section

### 5.1 Materials

PVA, SPS, and Ru were purchased from Sigma‐Aldrich and used as received. Dichloromethane was purchased from Aladdin. TGF‐ β, IGF‐1, and VEGF were bought from Novoprotein. HAMA‐150K was purchased from Engineering for Life (EFL). BSA‐FITC solution was obtained from Solarbio.

### 5.2 Structural Analysis of the PVA‐Tyr macromer via NMR

PVA‐Tyr macromer was gifted by Khoon S. Lim from Sydney University. The conjugation efficiency of tyramine groups to the PVA backbone was assessed by ^1^H NMR analysis. First, PVA‐Tyr samples were dissolved in deuterium oxide (D_2_O) and spectra were acquired on a Bruker Advance DPX‐300 spectrometer operating at 300 MHz. The percentage conjugation was calculated by comparing the integral of the aromatic proton signals (δ 6.5–7.5 ppm) to that of the protons from the PVA backbone. This value was then used to determine the number of crosslinkers per polymer chain with the equation:

$$\mathrm{Tyramine}\;\mathrm{groups}\;\mathrm{per}\;\mathrm{chain}\;=\frac{MW_{\mathrm{polymer}}}{MW_{\mathrm{RU}}}$$

MW denotes molecular weight, and RU stands for the repeating unit of the polymer.

### 5.3 Fabrication and Characterization of PVA‐Tyr MPs

Dried PVA‐Tyr macromer was dissolved in ultrapure water at 80 °C. Oil–water two‐phase emulsion method was used for the fabrication of PVA‐Tyr MPs. Briefly, stock solutions of photoinitiators (Ru/SPS) were added to PVA‐Tyr solution under light‐free conditions. The final concentration of PVA‐Tyr was 5 wt% with Ru/SPS concentration ranging from 0.4/4 to 0.8/8 mm. For protein loading, BSA or GF solutions were blended with the macromer solution before introducing Ru/SPS to the system. The mix was added to dichloromethane and the oil–water system was subjected to intense vortex mixing at 3500 rpm for 3 min to obtain MPs. The system was then irradiated under visible light from a LED flashlight (15 W) for another 3 min to induce the photo‐crosslinking process. Fabricated MPs will be rinsed with phosphate‐buffered saline (PBS) for two times and then subject to the direct use. Morphology and size of PVA‐Tyr MPs were characterized by optical microscope and Image J analysis.

### 5.4 Fabrication and Characterization of the MN patch

Dried HAMA was dissolved at 5 wt% in 0.25 wt% lithium phenyl‐2,4,6‐trimethylbenzoylphosphinate (LAP) solution in the 50 °C water bath until total dissolution. The HAMA solution (100 µL) with or without MPs was added to the custom‐fabricated negative polydimethylsiloxane (PDMS) MN molds (10 × 10 array) with 300 µm‐radius base and 550 µm length. The solution was vacuumed for at least 30 min to remove all the air bubbles and then be put into a 30 °C oven to dry until the liquid level approached the bottom surface of the mold. These steps were repeated for three times to reach a total volume of 300 µL HAMA. A plastic scraper was then used to wipe off the viscous HAMA solution above cavities. Subsequently, another volume of 100 µL PVA solution (20 wt%) was added to the mold and subject to drying until a layer of flexible base membrane formed. Polymerization was initiated by exposing the MN to the ultraviolet light source (UVSP81T, FUTANSI, 405 nm, 300 µW cm^−2^) for 30 s. MN patch could be detached readily from the mold after polymerization.

Morphology of the MN patch was observed by the optical microscope and SEM. Mechanical strength of the MN patch was determined by the Universal Testing Machine (Instron). A stainless‐steel plate was used to approach and compress the MN patch at a speed of 0.1 mm/s. The load capacity was set at 10 N and the initial distance was 2.0 mm.

### 5.5 Retention, Degradation, and Release Profiles

To determine the retention and degradation profiles of PVA‐Tyr MPs and release kinetics of MP loaded MNs, FITC labeled BSA was encapsulated into MPs as the model protein as per procedures in section *Fabrication and Characterization of PVA‐Tyr MPs*. MPs alone or MP loaded MNs (with MPs embedded within the MN tip cavities) were then immersed into 1 mL PBS at 37 °C. Fluorescence intensity of the supernatant was measured by microplate reader at selected time points. Fluorescence intensity of the supernatant control, which derived from the plain PVA‐Tyr MP or MP loaded MN, was set as the background. PBS was refreshed at each time point. Retention rate was calculated as the percentage of proteins reserved in MPs after rinses since rinses substantially mitigated burst release effects. Because MPs are confined inside MN tips, direct time‐resolved quantification of MP mass loss is impractical; therefore, degradation progression was quantified operationally using the fluorescence‐based cumulative release readout. Specifically, MP “degradation time” was defined as the time to reach complete release/MP disintegration after standardized rinses (i.e., 100% cumulative release under the test conditions). Degradation time of MPs was recorded as they achieved complete release (100% degradation). Cumulative release of MP loaded MN was calculated as summing time‐point‐specific fluorescence values, with normalization against the total fluorescence intensity at complete release.

### 5.6 Mathematical Analysis of Release Kinetics

To further interpret the cumulative release profiles mechanistically, the release process was analyzed using a two‐stage framework consisting of an initial burst phase and a post‐burst sustained phase. The burst fraction was defined from the cumulative release at day 1 as

$$B={\left(\frac{M_{t}}{M_{\infty }}\right)}_{t=1\mathrm{day}}$$

The residual post‐burst fraction was then normalized as

$$F_{\mathrm{rem}}\left(t\right)=\frac{M_{t}/M_{\infty }-B}{1-B}$$

To analyze the post‐burst phase, the time origin was shifted to day 1 (i.e., 𝑡′ = 𝑡 − 1 day), and the standard Weibull model was applied as

$$F_{\mathrm{rem}}\left(t^{\prime }\right)=1-\exp\left[-{\left(\frac{t^{\prime }}{\tau }\right)}^{\beta }\right]$$

where τ denotes the characteristic release time and 𝛽 denotes the shape parameter. This treatment should be understood as a shifted‐time application of the standard Weibull model, mathematically equivalent to the lag‐time Weibull forms used in recent release studies [86, 87].

The Weibull model was selected as the primary fitting model because recent literature supports its use for describing polymeric release profiles and interpreting release mechanisms through the 𝛽 parameter. By contrast, the Korsmeyer–Peppas model is classically applicable mainly to the first ∼60% of release and was therefore not used as the primary model for the whole profile in the present study [52, 53].

### 5.7 Fabrication of GF Loaded MPs for Both In Vitro and In Vivo Bioactivity Assays

Different GFs including TGF‐β, IGF‐1, and VEGF were individually encapsulated into MPs with different photoinitiator concentrations to achieve tailored degradation time (as operationally defined in the section above). Fabricated MPs were directly loaded into the MN and stored at −20 °C for further use. For in vitro experiments, GF MP MNs were immersed into the basal medium until complete release. For in vivo experiments, GF MP MN was attached to the infarction site after ligation. The three GF‐loaded MP populations were combined on an equal formulation basis (1:1:1), defined as the same input amount of PVA‐Tyr macromer used for synthesizing each MP population (equivalently, the same volume of 5 wt% PVA‐Tyr solution), rather than equal GF mass, equal molar amount, or equal particle number. Each MN patch was designed to contain a theoretical loading of 200 ng TGF‐β, 200 ng IGF‐1, and 200 ng VEGF before accounting for formulation retention. The three Ru/SPS conditions corresponded to measured release‐completion times of approximately 5, 15, and 29 days and were operationalized as nominal 7‐, 14‐, and 28‐day windows in the schedule‐control animal study. The 200 ng nominal loading was selected as a processability‐constrained, literature‐guided dose and was not intended to represent an exhaustively optimized three‐factor stoichiometry.

### 5.8 Cell Culture

RAW 264.7 cells and H9c2 cells were purchased from Guangzhou Cellcook Biotech Co., Ltd. (Guangzhou, China). Both cells were cultured in high‐glucose Dulbecco's modification of Eagle medium (DMEM; Servicebio, China) supplemented with 10% fetal bovine serum (FBS; Vivacell, China) and 1% penicillin/streptomycin (P/S; Servicebio, China). HUVECs were purchased from MEISEN CELL (Zhejiang, China) and cultured in complete Endothelial Cell Medium (ScienCell, USA).

### 5.9 Macrophage Polarization and qRT‐PCR Analysis

RAW 264.7 cells were seeded in 24‐well plates and cultured until 70%–80% confluence. Cells were stimulated with LPS (10 ng/mL) for 8 h (no FBS) and cocultured with empty MP MN lysates, free TGF‐β, or TGF‐β MP MN lysates, whereas the control group received no co‐culture treatment. Total RNA of RAW264.7 was extracted using the FastPure Cell/Tissue Total RNA Isolation Kit (Vazyme, China). RNA was then reverse transcripted to cDNA with HiScript IV RT SuperMix for qPCR (Vazyme, China). Taq Pro Universal SYBR qPCR Master Mix (Vazyme, China) was used for qRT‐PCR on the QuantStudio 3 Real‐Time PCR System (ThermoFisher). Primers were synthesized by Sangon Biotech (China). Primer sequences used for RAW264.7 cells were listed in Table S1 (Supporting Information).

### 5.10 Flow Cytometry Analysis of RAW264.7 Cell Phenotype

For phenotypic analysis by flow cytometry, RAW 264.7 cells were stimulated with LPS (10 ng/mL) for 24 h (no FBS) and cocultured with empty MP MN lysates, free TGF‐β, or TGF‐β MP MN lysates, whereas the control group received no co‐culture treatment. RAW264.7 cells were stained with CD86 (BD Pharmingen) and CD206 (Biolegend) antibodies. Briefly, Fc receptors of cells were blocked with anti‐mouse CD16/32 antibody (Biolegend) for 10 min at 4 °C. Cells were then incubated with CD86 antibody in the dark at room temperature for 30 min. Subsequently, fixation and permeabilization were performed using Fixation/Permeabilization Solution Kit (BD Pharmingen). Intracellular staining of CD206 was then conducted on cells in the dark at room temperature for 30 min. Data collection was performed on CytoFLEX S flow cytometer (Beckman coulter, USA), and the resulting data were analyzed with FlowJo software (version 10.8, BD, USA).

### 5.11 Nitric Oxide Production Assay

NO production was assessed by measuring the nitrite concentration in the culture supernatant via the Griess reaction. RAW264.7 cells were seeded in 96‐well plates and incubated overnight. Cells were then stimulated with LPS (10 ng/mL) for 24 h and cocultured with empty MP MN lysates, free TGF‐β, or TGF‐β MP MN lysates, whereas the control group received no co‐culture treatment. For the assay, 50 µL of supernatant was combined with 50 µL of Griess Reagent I and 50 µL of Griess Reagent II. The absorbance at 540 nm was measured immediately, and nitrite concentrations were calculated against a sodium nitrite standard curve.

### 5.12 Hypoxia Treatment of H9c2 Cells

Induction of physical hypoxia in H9c2 cells was achieved by utilizing a hypoxia incubator chamber (Billups Rothenberg). Briefly, the chamber was prewarmed and humidified at 37 °C for over 30 min before placing H9c2 cells into it. A gas mixture of 1% O_2_, 5% CO_2_, and 94% N_2_ was then used to flush the chamber to create a hypoxic environment. Subsequently, the chamber was sealed and transferred to the 37 °C incubator. H9c2 cells were maintained in basal medium and co‐cultured with empty MP MN lysates, free IGF‐1 (50 ng/mL), or IGF‐1 MP MN lysates, while the group that received no co‐culture served as the control.

### 5.13 Determination of Cell Viability

To evaluate the effects of IGF‐1 released from the hydrogel on cell viability, live/dead double staining (Calcein‐AM/PI) and CCK‐8 assay were conducted on H9c2 cells.

For live/dead double staining, cells were seeded in a 24‐well plate until 70%–80% confluence. After 24‐hour treatment, the medium was removed and cells were rinsed once with PBS. Cells were treated with Calcein‐AM/PI working solution and incubated at 37 °C for 30 min in the dark. Fluorescence microscopy was then used to observe the characteristic green fluorescence of live cells (Calcein‐AM) and red fluorescence of dead cells (PI). Image J software (National Institutes of Health, USA) was employed for quantification.

For CCK‐8 assay, cells were seeded in a 96‐well plate until 70%–80% confluence. After 24‐h treatment, 10 µL of CCK‐8 solution was added to each well, which was followed by a 1 h incubation at 37 °C. Absorbance was subsequently measured at 450 nm using a Microplate Reader (SpectraMax iD5, Molecular Devices, USA).

### 5.14 Determination of Cell Apoptosis

Cell apoptosis was assessed with JC‐1 staining, flow cytometric analysis (Annexin V‐FITC/PI), and TUNEL assay.

The mitochondrial membrane potential (ΔΨm) of H9c2 cells was assessed using the JC‐1 fluorescent probe, following the manufacturer's instructions. Briefly, cells were stained with a 5 µm JC‐1 solution for 30 min at 37 °C after different treatments. Following two washes with PBS, the fluorescence was visualized under a microscope (ICX41, Sunny Optical, China). In cells with healthy, high ΔΨm, JC‐1 forms aggregates that emit red fluorescence, whereas in cells with depolarized mitochondria, it remains in a monomeric state emitting green fluorescence.

For flow cytometric analysis of apoptosis, H9c2 cells from different treatment groups were collected, washed with cold PBS, and resuspended in binding buffer. Cells were then stained with Annexin V‐FITC and PI per the manufacturer's protocol (Annexin V‐FITC Apoptosis Detection Kit, Beyotime, China) and subject to flow cytometry using a CytoFLEX S flow cytometer (Beckman Coulter, USA) to identify apoptotic populations. Data processing was performed with FlowJo software (version 10.8, BD, USA).

For TUNEL assay, cells from each treatment group were fixed with 4% paraformaldehyde and permeabilized in 0.1% Triton X‐100, which was followed by labeling of apoptotic cells using a TUNEL Apoptosis Detection kit (Servicebio, China). Nuclei were counterstained with DAPI, and fluorescence imaging was obtained with a fluorescence microscope (ICX41, Sunny Optical, China).

### 5.15 Wound Healing Assay

Wound healing assay was used to evaluate the effects of VEGF released from the hydrogel on migration of HUVECs. HUVECs were seeded in 24‐well plates and cultured to confluence. Cells were then subject to vertical scratch with a 200 µL pipette tip. Cell debris was removed by wash with PBS for two times. HUVECs were incubated in basal Endothelial Cell Medium supplemented with 1% FBS and cocultured with empty MP MN lysates, VEGF (10 ng/mL), and VEGF MP MN lysates, respectively; the group that received no coculture was used as the control. Images of wound area in each well were captured with an inverted microscope at 0 and 24 h. Image J software was used to measure the wound area. To quantify the migration ability of HUVECs, cell migration percentage was calculated according to the following equation: Cell migration percentage (%) = [(*A*_0_ − *A*_1_)/*A*_0_] × 100%, where *A*_0_ represents the initial area and *A*_1_ represents the remaining area after treatment for 24 h.

### 5.16 Tube Formation Assay

GF reduced matrigel (Corning, USA) was incubated at 4 °C overnight until complete thawing. It was then mixed with basal Endothelial Cell Medium at the ratio of 1:1. Each well of the 96‐well plate was coated with 50 µL mixture and the plate was placed at 37 °C for 1 h to solidify the mixture. HUVECs (1 × 10^4^ cells/well) were then seeded onto the solidified matrigel and incubated with basal Endothelial Cell Medium containing empty MP MN lysates, VEGF (10 ng/mL), and VEGF MP MN lysates, respectively; the group that incubated with basal Endothelial Cell Medium only was used as the control. After incubation for 12 h at 37 °C under 5% CO_2_, tube networks were photographed with an inverted microscope. Image J software was used for the quantification.

### 5.17 Proliferation Assay

HUVECs were inoculated in 24‐well plates at the density of 1 × 10^4^ cells/well and incubated in the complete Endothelial Cell Medium overnight until complete cell adhesion. Cells were then gently washed with PBS for two times and incubated in basal Endothelial Cell Medium containing 5% FBS. Different treatments were applied to HUVECs, including empty MP MN lysates, VEGF (10 ng/mL), and VEGF MP MN lysates, respectively; the group that received no coculture was used as the control.

### 5.18 Rat MI Model Establishment and MN Treatment

All animal experiments were conducted in accordance with the guidelines of the ethics committee of the Experimental Animal Center of the Chinese University of Hong Kong, Shenzhen (CUHKSZ–AE2024013). Male SD rats (200–220 g, 6–7 weeks old) were purchased from the Charles River. All rats were subject to acclimation for 1 week before the experiment. MI model was established as previously reported [88]. Briefly, rats were anesthetized via intraperitoneal injection of sodium pentobarbital (1%w/v) and then subject to ventilation. A left thoracotomy was performed to expose the heart. MI was obtained by permanent ligation of the left anterior descending (LAD) coronary artery using a 6‐0 silk suture. Different MN patches were then be attached to the infarct area. Seven treatments were applied to rats randomly (*n* = 6): (a) Sham (rats only received a thoracotomy); (b) MI control (MI rats experienced no further treatments); (c) MI + empty MP MN (MP patch loaded with empty MPs); (d) MI + TGF‐β MP MN (MN patch filled with TGF‐β loaded MPs); (e) MI + IGF MP MN (MN patch filled with IGF‐1 loaded MPs); (f) MI + VEGF MP MN (MN patch filled with VEGF loaded MPs); (g) MI + GF MP MN (MN patch filled with TGF‐β/IGF‐1/VEGF loaded MPs).

### 5.19 Additional Schedule‐Control Animal Study

An independent schedule‐control cohort included Sham, MI, simultaneous completion within 7 days, simultaneous completion within 28 days, VEGF/TGF‐β/IGF‐1 completion within 7/14/28 days, IGF‐1/VEGF/TGF‐β completion within 7/14/28 days, and the original TGF‐β/IGF‐1/VEGF 7/14/28‐day program (*n* = 6 per group). In all treatment groups, each GF was separately encapsulated in PVA‐Tyr MPs, and the resulting MP populations were loaded into the MNs. The release windows were programmed through the Ru/SPS concentration used during MP synthesis.

### 5.20 Echocardiographic Evaluation of Cardiac Function

Echocardiography was performed to assess the LV function prior to MI surgery as baseline and on day 7 and 28 postsurgery. Isoflurane was used for anesthesia of rats and echocardiography tests were then performed with the VINNO 6LAB ultrasound system. Two‐dimensional (2D) mode and 2D‐guided M‐mode images were captured for the subsequent analysis of cardiac parameters.

### 5.21 Histological Analysis

Hearts were harvested at day 7 and day 28 after MN treatment. They were fixed in 4% paraformaldehyde and subjected to paraffin embedding. Embedded hearts were cut into sections with thickness of 5 µm. Masson, Sirius red, and immunofluorescence staining were conducted on samples according to the standard protocol to evaluate the effects of MN treatment on inflammation, apoptosis, infarction size, fibrosis, and vascularization.

### 5.22 qRT‐PCR Analysis of Myocardial Tissues

MN attached myocardial tissues were grinded to thorough homogenization with a high‐speed Tissue Homogenizer (Servicebio, China). Total RNA extraction of heart tissues, transcription to cDNA, and qRT‐PCR can be referred to the section “Macrophage Polarization and qRT‐PCR Analysis”. Primer sequences used for myocardial tissues were synthesized by Sangon Biotech (China) and listed in Table S2.

### 5.23 RNA‐seq Analysis

For RNA‐seq of MI tissues, 7 and 28 days post‐MI, rats were euthanized and MI tissues were then rapidly harvested. Obtained tissues were preserved in RNAsolid RNA Stabilization Solution for Tissue (Servicebio, China). Total RNA extraction of both cells and tissues utilized same steps mentioned in “Macrophage Polarization and qRT‐PCR Analysis” and “qRT‐PCR Analysis of Myocardial Tissues”. The mRNA was isolated from total RNA via column purification and served as the primary material for high‐throughput RNA sequencing (BGI, China).

### 5.24 Statistical Analysis

Data analysis was performed using GraphPad Prism 10. All data were presented as mean ± standard deviation. One‐way analysis of variance (ANOVA) followed by Tukey's test was used to compare the variance among more than two groups. Significance was defined as: ns (non‐significant), * (*p* < 0.05), ** (*p* < 0.01), *** (*p* < 0.001), and **** (*p* < 0.0001).

### 5.25 Terminology

PVA‐Tyr macromer: Uncrosslinked tyramine‐modified poly(vinyl alcohol) precursor; PVA‐Tyr MPs/MPs: Crosslinked PVA‐Tyr hydrogel microparticles prepared from the PVA‐Tyr macromer by vortex‐assisted oil/water emulsification followed by Ru/SPS‐mediated visible‐light crosslinking; Bulk PVA‐Tyr hydrogel: Non‐emulsified crosslinked hydrogel prepared from the same PVA‐Tyr macromer/Ru/SPS chemistry.

## Author Contributions

The research was conceptualized by J.T. and X.C. The methodology was developed by Y.D., P.D., and Z.W. Experiments were conducted by Y.D., P.D., Z.W., Surilige, R.F., X.Z., S.Z., and R.G. Visualization was handled by Y.D. and X.C. Supervision was provided by J.T. and X.C. Y.D. and X.C. were responsible for writing, while Y.D., R.T., Y.W., Y.Z., Z.F., C.J., Y.H., Q.W., R.L., K.L., J.T., and X.C. contributed to review and editing.

## Conflicts of Interest

X.C., Y.D., and Z.W. have filed a patent based on the outcome of this study.

## Supporting information




**Supporting File 1**: advs77101‐sup‐0001‐SuppMat.docx.

**Supporting File 2**: advs77101‐sup‐0002‐VideoS1.mp4.

## Data Availability

The data that support the findings of this study are available from the corresponding author upon reasonable request.
