# Supplementary material for: Programmable Paracrine‐Mimetic Microneedle System for Temporal Regulation of Cardiac Repair
Source: Adv Sci (Weinh). 2026 Aug 18:e77101. Online ahead of print. doi: 10.1002/advs.77101 (PMC13482941; doi:10.1002/advs.77101)
Supplement: Supplementary file 1 [file ADVS-9999-e77101-s001.docx]

Supplementary Materials

**Programmable Paracrine-Mimetic Microneedle System for Temporal Regulation of Cardiac Repair**

Yichen Dai^1,2#^, Pengchong Du^3,4,5,6#^, Zhaoyang Wang^1,2#^, Surilige^3,4,5,6#^, Rangga Fokta^1,2^, Renjian Tan^7^, Xiqing Zhao^3,4,5^, Shuoyi Zhang^1^, Yuecheng Wang^1,2^, Yixin Zhang^1,2^, Ziliang Fu^1,2^, Rui Gong^1,2^, Cheng Jiang^1^, Yisheng He^1^, Qiguang Wang^8^, Renke Li^1^, Khoon Lim^7*^, Junnan Tang^3,4,5,6^*, Xiaolin Cui^1,2^*

1. School of Medicine, The Chinese University of Hong Kong, Shenzhen, Guangdong, 518172 China

2. CUHKSZ-Dalian Practical Biotechnology Joint Laboratory for Biomedicine, The Chinese University of Hong Kong, Shenzhen, Guangdong, 518172 China

3. Department of Cardiology, The First Affiliated Hospital of Zhengzhou University, Henan 450000, China

4. Henan Province Key Laboratory of Cardiac Injury and Repair, Zhengzhou, Henan 450052, China

5. Henan Province Clinical Research Center for Cardiovascular Diseases, Zhengzhou, Henan 450052, China

6. State Key Laboratory of Metabolic Dysregulation & Prevention and Treatment of Esophageal Cancer, Tianjian Laboratory of Advanced Biomedical Sciences, Academy of Medical Sciences, Zhengzhou, Henan 450001, China.

7. School of Medical Sciences, University of Sydney, NSW, 2001, Australia

8. College of Biomedical Engineering, Sichuan University, Chengdu, Sichuan 610065, China

Corresponding authors

Khoon Lim: khoon.lim@sydney.edu.au

Junnan Tang: fcctangjn@zzu.edu.cn

Xiaolin Cui: stevencui@cuhk.edu.cn

YD, PD and ZW contributed equally to this work

**Supplementary methods**

*Additional in vitro evaluation of the combined GF/GF MP MN formulation in RAW264.7 macrophages*: RAW 264.7 cells were seeded in 24-well plates and cultured until 70-80% confluence. Cells were stimulated with LPS (10 ng/mL) for 8 hours (no FBS) and cocultured with empty MP MN lysates, free GF cocktail, or GF MP MN lysates, whereas the control group received no co-culture treatment. The free GF cocktail contained TGF-β, IGF-1, and VEGF. Total RNA of RAW264.7 was extracted using the FastPure Cell/Tissue Total RNA Isolation Kit (Vazyme, China). RNA was then reverse transcripted to cDNA with HiScript IV RT SuperMix for qPCR (Vazyme, China). Taq Pro Universal SYBR qPCR Master Mix (Vazyme, China) was used for qPCR on the QuantStudio 3 Real-Time PCR System (ThermoFisher). Primers were synthesized by Sangon Biotech (China). Primer sequences used for RAW264.7 cells were listed in **Table S1**.

*Additional in vitro evaluation of the combined GF/GF MP MN formulation in H9c2 cells*: H9c2 cells were cultured to 70–80% confluence and then subjected to hypoxic treatment (1% O₂) to simulate ischemic conditions. The following groups were included: normoxia blank, hypoxia blank, hypoxia + empty MP MN lysates, hypoxia + free GF cocktail, and hypoxia + GF MP MN lysates.

The mitochondrial membrane potential (ΔΨm) of H9c2 cells was assessed using the JC-1 fluorescent probe, following the manufacturer's instructions. Briefly, cells were stained with a 5 μM JC-1 solution for 30 minutes at 37 °C after different treatments. Following two washes with PBS, the fluorescence was visualized under a microscope (ICX41, Sunny Optical, China). In cells with healthy, high ΔΨm, JC-1 forms aggregates that emit red fluorescence, whereas in cells with depolarized mitochondria, it remains in a monomeric state emitting green fluorescence. Cell viability was assessed using the CCK-8 assay according to the manufacturer’s instructions.

*Additional in vitro evaluation of the combined GF/GF MP MN formulation in HUVECs*: HUVECs were used to further evaluate the broad biological activity of the combined GF/GF MP MN formulation. For the wound healing assay, HUVECs were seeded in 24-well plates and cultured to confluence. A vertical scratch was made using a 200 μL pipette tip, and detached cells were removed by washing with PBS. Cells were then cultured in basal medium supplemented with 1% FBS and treated with blank control, empty MP MN lysates, free GF cocktail, or GF MP MN lysates. The free GF cocktail contained TGF-β, IGF-1, and VEGF. Images of the wound area were captured at 0 and 24 h, and the wound closure area was quantified using ImageJ software. Cell migration percentage was calculated according to the following equation: Cell migration percentage (%) = [(A_0_ – A_1_)/ A_0_] × 100%, where A_0_ represents the initial area and A_1_ represent the remaining area after treatment for 24 h.

For the proliferation assay, HUVECs were seeded in 24-well plates and cultured overnight until complete cell adhesion. Cells were then washed with PBS and incubated in basal endothelial cell medium containing 5% FBS. The following treatment groups were applied: blank control, empty MP MN lysates, free GF cocktail, and GF MP MN lysates. Cell proliferation was assessed using the Alamar Blue assay according to the manufacturer’s instructions.

*Statistical analysis*: All experiments were conducted in triplicate unless otherwise indicated. GraphPad Prism was used for the analysis. All data will be shown as means ± standard deviation (SD). One-way analysis of variance (ANOVA) followed by Tukey’s test was used to compare the variance among more than two groups.

**Supplementary results**

***Additional supportive in vitro validation of the combined GF/GF-MP formulation across multiple relevant cell types***

To further address whether the combined GF formulation retained broad biological activity beyond the canonical single-factor/cell-type validation experiments, additional *in vitro* studies were performed in multiple relevant cell models. In LPS-stimulated RAW264.7 macrophages, the combined GF MP MN treatment significantly reduced the expression of iNOS, TNF-α, IL-1β, and IL-6 compared with the LPS and empty MP groups, indicating preserved anti-inflammatory activity of the combined formulation (**Figure S9**). In hypoxia-treated H9c2 cells, the combined GF-MP formulation significantly improved cell viability in the CCK-8 assay. Collectively, these data provide supportive evidence that the combined GF-MP formulation retains broad biological activity across inflammation-, angiogenesis-, and cardiomyocyte-relevant *in vitro* settings (**Figure S10**). In HUVECs, the combined GF MP MN formulation significantly enhanced cell proliferation, as measured by the Alamar Blue assay, and promoted cell migration in the wound healing assay (**Figure S11**).

**Figure S1.** a) ¹H NMR characterization of the PVA-Tyr macromer; b) Mechanical strength of the MN patch; c) Masson trichrome staining of penetration sites; d) Storage modulus (G’), loss modulus (G’’), and gelation time of the bulk PVA-Tyr hydrogel prepared at 5 wt% PVA-Tyr with different Ru/SPS concentrations (0.4/4 mM, 0.6/6 mM, and 0.8/8 mM). Panel b corresponds to the mechanical strength of the HAMA-based MN patch, whereas panel d characterizes the PVA-Tyr chemistry relevant to MP formation and degradation.

**Figure S2.** a) SEM image of the PVA-Tyr MP; b) degradation time of PVA-Tyr MPs with different Ru/SPS concentrations (0.4/4 mM, 0.6/6 mM, 0.8/8 mM, 1/10 mM, 2/20 mM) (n = 3)

**Figure S3.** Burst-separated Weibull fitting of the cumulative release profiles of BSA from MNs loaded with PVA-Tyr MPs. Symbols represent experimental data; solid lines represent burst-separated Weibull fits. MPs were prepared with different Ru/SPS concentrations (0.4/4 mM, 0.6/6 mM, and 0.8/8 mM). (n = 3) The burst fraction at day 1 was quantified experimentally, and the post-burst phase was fitted using the standard Weibull model after redefining the time origin at day 1.

**Figure S4.** RNA-seq analysis of RAW264.7. a) Hierarchical clustering of differentially expressed genes in LPS and TGF-β MP groups (|Log2Fold Change| ≥ 1, p-Value < 0.05); b) GSEA analysis of differential genes and enrichment plots of the correlative pathways (n = 3).

**Figure S5.** Apoptosis rate of H9c2 *in vitro* evaluated by Annexin V/PI staining and flow cytometry analysis.

**Figure S6.** IGF-1 released from PVA-Tyr MPs reduced apoptosis rate of H9c2 in vitro. a) Representative images of TUNEL staining and b) quantification (n = 6). Scale bar = 100 μm.

**Figure S7.** RNA-seq analysis of H9c2. a) Hierarchical clustering of differentially expressed genes in Hypoxia and IGF MP groups (|Log2Fold Change| ≥ 1, p-Value < 0.05); b) GSEA analysis of differential genes and enrichment plots of the correlative pathways (n = 3).

**Figure S8.** RNA-seq analysis of HUVECs. a) Hierarchical clustering of differentially expressed genes (|Log2Fold Change| ≥ 1, p-Value < 0.05); b) GSEA analysis of differential genes and enrichment plots of the correlative pathways (n = 3).

**Figure S9.** qRT-PCR analysis of inflammatory gene expression in LPS-stimulated RAW 264.7 cells after treatment with the combined GFs released from PVA-Tyr MP MN. (n = 6)

**Figure S10.** GFs released from PVA-Tyr MP MN promoted survival of H9c2 in vitro. a) Representative image of JC-1 fluorescence staining for the changes in mitochondrial membrane potential (ΔΨm) and b) quantitative analysis (n = 3); c) Relative viability of cells measured by CCK-8 assay (n = 6). Scale bar = 200 μm.

**Figure S11.** Effects of combined GFs released from PVA-Tyr MP MN on HUVECs. a) migration and b) quantification, and c) proliferation profile (n = 6). Scale bar = 200 μm

**Figure S12.** Attachment of the MN patch on MI rat hearts.

**Figure S13.** Sirius red staining of infracted sites after different treatments for 4 weeks.

**Figure S14.** RNA-seq analysis of MI and GF MP MN groups at day 28. a) Hierarchical clustering of differentially expressed genes (|Log2Fold Change| ≥ 1, p-Value < 0.05); b) GSEA analysis of differential genes and enrichment plots of the correlative pathways.

**Figure S15.** RNA-seq analysis of Empty MP MN and MI groups at day 28: a) Volcano plot of differentially expressed genes between Empty MP MN and MI groups (|Log2Fold Change| ≥ 1, p-Value < 0.05); b) GO pathway-enrichment analysis of differential genes between Empty MP MN and MI groups; c) GSEA analysis of differential genes and enrichment plots of the most correlative pathways (n = 3); d) KEGG pathway-enrichment analysis of differential genes between Empty MP MN and MI groups.

**Figure S16.** RNA-seq analysis of GF MP MN and Empty MP MN groups at day 28: a) Volcano plot of differentially expressed genes between GF MP MN and Empty MP MN groups (|Log2Fold Change| ≥ 1, p-Value < 0.05); b) GO pathway-enrichment analysis of differential genes between GF MP MN and Empty MP MN groups; c) GSEA analysis of differential genes and enrichment plots of the most correlative pathways (n = 3); d) KEGG pathway-enrichment analysis of differential genes between GF MP MN and Empty MP MN groups.

**Figure S17.** *In vivo* effects of GF MP MNs on serum level of TNF-α, IL-1β, and MCP-1 (n = 3).

**Figure S18.** Representative M-mode echocardiographic images showing the effects of different triple-GF release schedules on cardiac function at day 28 after MI (n = 6). The tested schedules were Sham, MI, simultaneous release within 7 days, simultaneous release within 28 days, VEGF/TGF-β/IGF-1 release within 7/14/28 days, IGF-1/VEGF/TGF-β release within 7/14/28 days, and the sequential release program (original GF MP MN group). The corresponding quantitative analyses of LVEF, LVFS, ΔLVEF, and ΔLVFS are shown in **Figure 9a**.

**Figure S19.** Representative Masson trichrome-stained sections and enlarged images of the infarct region showing the effects of different triple-GF release schedules on infarct-wall remodeling at day 28 after MI (n = 6). Scale bars = 4 mm (heart sections) and 500 μm (enlarged images). The release schedules are defined in **Figure S18**. The corresponding quantitative analyses of infarct-wall thickness and collagen volume fraction are shown in **Figure 9b**.

**Table S1.** Primer sequences for investigated genes of RAW 264.7 cells.

| Gene | Forward primer | Reverse primer |
| --- | --- | --- |
| *iNOS* | GAGACAGGGAAGTCTGAAGCAC | CCAGCAGTAGTTGCTCCTCTTC |
| *TNF-α* | GCCTCTTCTCATTCCTGCTTGTGG | GTGGTTTGTGAGTGTGAGGGTCTG |
| *IL-1β* | TCGCAGCAGCACATCAACAAGAG | AGGTCCACGGGAAAGACACAGG |
| *IL-6* | CTTCTTGGGACTGATGCTGGTGAC | AGGTCTGTTGGGAGTGGTATCCTC |
| *Gapdh* | ACGGCAAATTCAACGGCACAG | ACACCAGTAGACTCCACGACATAC |

**Table S2.** Primer sequences for investigated genes of myocardial tissues.

| Gene | Forward primer | Reverse primer |
| --- | --- | --- |
| *Collagen Ⅰ* | GGAAACCTGATGTATGCTTGAT | GACTCCTATGACTTCTGCGTCT |
| *Collagen Ⅲ* | TGGATTTCAAGATCAACACTGA | TCCGGTTTCCATATTACAGAAC |
| *α-SMA* | CATCAGGAACCTCGAGAAGC | TCGGATACTTCAGGGTCAGG |
| *VEGFr2* | GCCTTATGATGCCAGCAAGT | ACTTGCTGGCATCATAAGGC |
| *TNF-α* | GCATGATCCGAGATGTGGAA | CAGACACCGCCTGGAGTTCT |
| *TGF-β* | GCAACAATTCCTGGCGTTACC | TTCCGTCTCCTTGGTTCAGC |
| *iNOS* | TTTGGCCCGAAGGTCGC | AGGGATTCTGGAACATTCTGTG |
| *Gapdh* | CAGGGCTGCCTTCTCTTGTG | AACTTGCCGTGGGTAGAGTC |
